# Structural transitions in the RNA 7SK 5′ hairpin and their effect on HEXIM binding

**DOI:** 10.1093/nar/gkz1071

**Published:** 2019-11-16

**Authors:** Konstantin Röder, Guillaume Stirnemann, Anne-Catherine Dock-Bregeon, David J Wales, Samuela Pasquali

**Affiliations:** 1 Department of Chemistry, University of Cambridge, Lensfield Road, Cambridge CB2 1EW, UK; 2 CNRS Laboratoire de Biochimie Théorique, Institut de Biologie Physico-Chimique, PSL University, Université de Paris, 13 rue Pierre et Marie Curie, 75005 Paris, France; 3 Laboratoire de Biologie Intégrative des Modèles Marins, UMR CNRS 8227, Sorbonne Université, Station Biologique de Roscoff, 29680 Roscoff, France; 4 Laboratoire CiTCoM, CNRS UMR 8038, Université de Paris, 4 Avenue de l'observatoire, 75270 Paris, France

## Abstract

7SK RNA, as part of the 7SK ribonucleoprotein complex, is crucial to the regulation of transcription by RNA-polymerase II, via its interaction with the positive transcription elongation factor P-TEFb. The interaction is induced by binding of the protein HEXIM to the 5′ hairpin (HP1) of 7SK RNA. Four distinct structural models have been obtained experimentally for HP1. Here, we employ computational methods to investigate the relative stability of these structures, transitions between them, and the effects of mutations on the observed structural ensembles. We further analyse the results with respect to mutational binding assays, and hypothesize a mechanism for HEXIM binding. Our results indicate that the dominant structure in the wild type exhibits a triplet involving the unpaired nucleotide U40 and the base pair A43-U66 in the GAUC/GAUC repeat. This conformation leads to an open major groove with enough potential binding sites for peptide recognition. Sequence mutations of the RNA change the relative stability of the different structural ensembles. Binding affinity is consequently lost if these changes alter the dominant structure.

## INTRODUCTION

7SK RNA is an essential component of the human 7SK ribonucleoprotein (snRNP) (1,2), a complex containing two additional core proteins, LARP7 and MePCE (3–5). Around 20,000 7SK RNP nuclear particles are found in mammalian cells (6). Not only is this abundance remarkable, but RNA 7SK has also been found in a range of organisms, including rodents, birds and amphibians, with a high degree of sequence conservation (7–12). The abundance and evolutionary conservation hint at a significant biological role for both snRNP and 7SK RNA. Indeed, in higher eukaryotes, the regulation of transcription by RNA-polymerase II is aided by RNA 7SK (2,13,14). In this process, the RNA 7SK is involved in the control of the positive elongation factor, P-TEFb, which regulates the transcription elongation phase (14). The association of RNA 7SK with P-TEFb leads to a down-regulation of P-TEFb, which results in transcription pauses (14–16). The association of RNA 7SK and P-TEFb requires binding of the protein HEXIM, which then binds to P-TEFb (17,18). One of the key features of infection with the human immunodeficiency virus (HIV) is hijacking of P-TEFb to elevate the transcription of the virus, so P-TEFb functionally links HIV and 7SK RNA (19). Indeed, a further similarity is found between HEXIM, the 7SK effector, and Tat, the HIV protein that triggers HIV transcription by binding to a structure named TAR located in the 5′ region of the HIV RNA. Both HEXIM and Tat use arginine-rich sequence motifs (ARM) to bind to RNA (20,21). However, the effect on the P-TEFb function is different, as it is inhibited by HEXIM-7SK and enhanced by Tat-TAR.

While alternative 2D structures for the RNA have been proposed (1,12), they consistently conserve two hairpins at the termini (11). Both hairpins contribute to the function of 7SK (22), with the 3′-hairpin involved in P-TEFb and LARP7 binding (22–26), and the 5′-hairpin playing an essential part in HEXIM recognition (22–24,27). The 5′-hairpin, HP1, consisting of nucleotides 24–87 in humans (12,28), also binds to the HIV trans-activator protein Tat in infected cells (29), which is responsible for the capture of P-TEFb (30).

Recently, a shortened version of HP1 (HP1-UUCG) was introduced (28) to facilitate crystallization, by replacing the large apical loop (nucleotides 49–59) by a stable tetraloop UUCG. This change does not impact the 7SK-motif, which comprises U(U)GAUC repeats, forming a short helix of four base-pairs framed by single-stranded uridines in the apical half of the HP1 hairpin (11,12,28). This motif is required for HEXIM binding (22,23,28,29).

**Figure 1.**
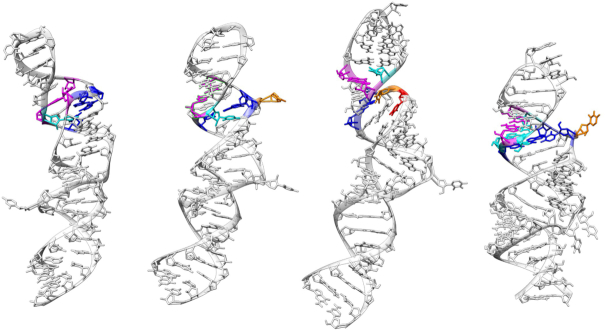
Representation of the four experimental structures from their respective PDB files, highlighting in colours the main differences in the 7SK motif. Exp1: U63 (cyan) is unpaired, U40–C45–G64 (magenta) forming a triplet, U41-A43-U66 (blue) forming a second triplet. Exp2: U63 (cyan) is unpaired, U41 (orange) is unpaired, U40–A43–U66 (blue) form a triplet. Exp3: U63 (cyan) is unpaired. U40 (red) and U41 (orange) are also unpaired. No triplets. Exp4: U41 (orange) is unpaired. U63–U44–A65 (cyan) form a triplet, U40–A43–U66 (blue) also form a triplet. (33).

Crystallographic (28) and NMR (31,32) investigations of the HP1-tetraloop hairpin revealed four conformations, two from crystallography, coexisting in the same crystal, and two from independent NMR experiments (Figure 1). The structures are immediately differentiated by their compactness, which results from specific intramolecular interactions. In the following text, Exp1 refers to the model IN and Exp2 to model OUT from the crystal structures (28) (PDB id: 5LYU), Exp3 is the more extended NMR structure (23) (PDB id: 5IEM), and Exp4 is the recently determined NMR structure (32) (PDB id: 6MCI), which is compact and has a very similar secondary structure organisation as Exp2, but with subtle differences that will be detailed in the next section. The tetraloop in this last structure has a different sequence, GAGA.

Additional investigation using molecular dynamics (MD) simulations suggested that Exp1 and Exp2 are stable conformations under physiological conditions (28). However, it is not clear how their stability compares with the models obtained from NMR, especially the extended model 3, how the transitions between the observed conformations occur, and how these transitions may relate to function. Several mutations of the sequence affect the HEXIM binding affinity (28), and these changes need to be better understood at the dynamical level. Indeed, the present understanding of RNA recognition by HEXIM is that while it relies, as most recognition events, on the establishment of specific hydrogen bonds and on π-stacking interactions, it also depends on the RNA conformational dynamics, which facilitate these interactions. Interestingly, recent work on the full-length 7SK RNA also emphasized the contribution of dynamics to function (34).

The hairpin HP1 also contains the binding site for Tat, and the recent NMR structure of the hairpin (Exp4) by Pham *et al.* was solved in parallel with a bound Tat peptide fragment, providing new insight into the recognition mechanism (32) (PDB id: 6MCF). Surprisingly, the structure with the bound peptide is very similar to the one without (6MCI), which could imply that the binding site is pre-formed. However, the observation of the different conformations Exp1–3 rather suggests a significant role of the RNA dynamics. Because of the similarity between the bound and unbound NMR structures, in the following discussion we will refer to these NMR structures simply as Exp4, without further distinction.

A theoretical framework for understanding such features in biomolecules is provided by the energy landscape, which contains all the information necessary to define kinetic, thermodynamic and structural properties. An exploration of the energy landscape can therefore provide a description of the system in atomistic detail, including very slow and very fast processes currently inaccessible to experiment. This approach embraces multifunnel landscapes (35), including detailed studies of mutational changes (36), and has been applied successfully to a range of nucleic acids, for example, to study the transformation between different helical DNA configurations (37,38), G-quadruplexes (39) and the formation of mini-dumbells in DNA (40).

In the present work, we aim to understand the role of the four different experimental structures with respect to the binding of HEXIM, and to develop an underlying model of operation for HP1 that connects all experimental evidence related to structures and mutations. We employ the computational potential energy landscape framework (41) to analyse the apical portion of HP1, containing the 7SK motif, for the native sequence and the mutations probed by Martinez-Zapien *et al.* (28) and to investigate the binding between HP1 and an arginine-rich motif (ARM) from HEXIM (20,23,42). Based on our results, we propose a regulation mechanism for HEXIM binding to HP1, which is consistent with experiment. Further, we elucidate the role of different nucleotides in this process, providing an interpretation of experimental results.

## MATERIALS AND METHODS

The structural transitions between the various experimental structures, as well as their correlations with the binding of HEXIM, have been studied through a number of complementary simulation techniques.

### 7SK structural ensemble

As indicated in the introduction, experimental work was conducted for a sequence with a loop modification of the hairpin, leading to the observation of four distinct structures (Exp1, Exp2, Exp3 and Exp4). Here, since we want to focus on the mechanism involving the 7SK binding motif (23), we chose an even shorter version, focusing on the top half of the hairpin, including nucleotides 37 to 70. Our motivation for this approach is the computational cost associated with exploring the energy landscape, especially as our study also aims to investigate the effects of mutations. The chosen sequence still contains the 7SK motif and the characteristic bonding patterns associated with the experimental structures, as we discuss below. However, the shortening will reduce our ability to discuss the full binding mechanism; it might introduce artefacts into the simulation, and may ignore other factors. Simulations of the full length HP1-UUCG (24–87), presented in Supplementary Material, highlight the rigidity of HP1-UUCG, with the top and the bottom stems never coming into contact, providing further justification for considering the upper portion separately from the rest, at least in this stage of our study.

For the shorter sequence, we introduce structural models that are derived from the experimentally observed structures and share their key characteristics. The two crystal structures, Exp1 and Exp2, are represented by our simulation models M1 and M2, respectively. For the NMR structure Exp3, we introduce the structural model E. As Exp4 was published after we completed our simulations, we have not included it in the initial stage. M1, M2 and E therefore represent families of structures all sharing some key structural features. In all models, the three 3′ and 5′ terminal nucleotides of the RNA form stable base pairs, C37–G70, C38–G69 and A39–U68, which prevent unfolding.

**Figure 2.**
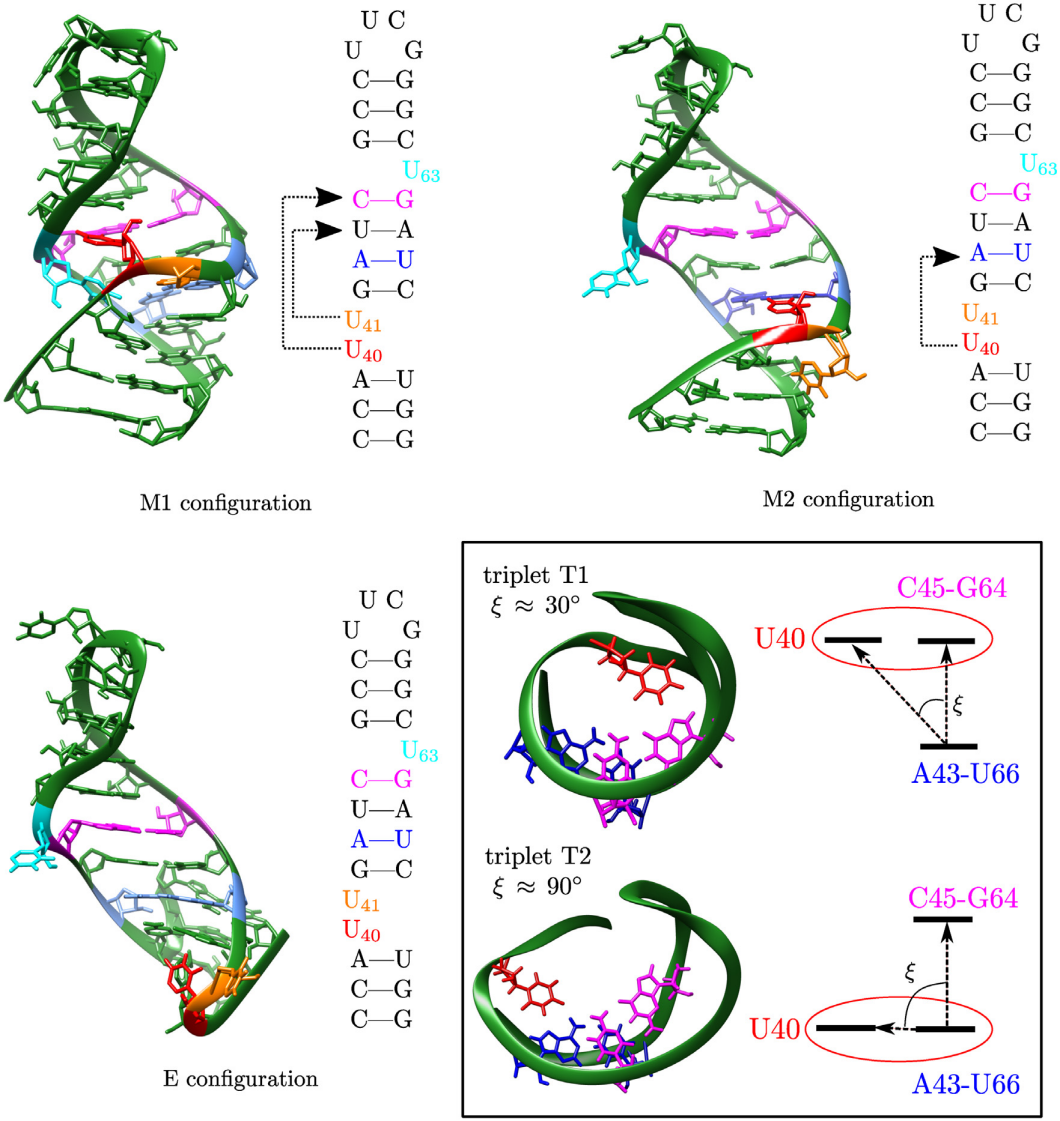
Example structures for the M1, M2 and E configurations with 2D representations highlighting the triplets formed (dashed arrows). The key residues in the hairpin are coloured in both the 2D and 3D representations. The same colouring scheme is used throughout the article. In the box in the bottom right hand corner the two distinct triplets formed by U40, triplet T1 and T2, are shown. The scheme on the right indicates how the angle ξ can differentiate between the two triplets, and subsequently between the M1 (triplet T1 formed) and M2 configurations (T2 triplet formed). In the E configuration no triplets are formed and ξ tends to 180°. The structures are taken from the wild type energy landscape as presented in Figure 4, and the 3D structural representations have been created using the UCSF Chimera package (33).

The M1 and M2 models are characterized by the formation of specific triplets (see Figure 2). In M1, two triplets are formed, pulling the top and bottom of the structure together, providing a very compact configuration. The triplets formed are U40–C45–G64 (triplet T1) and U41–A43–U66 (triplet T3). The three unpaired uracils, U40, U41 and U63, framing the GAUC/GAUC helix are not exposed to solvent. They are buried in the fold, and block access to the groove of the small helix of the 7SK-motif.

M2 is more extended, with only one triplet interaction, U40–A43–U66 (triplet T2). U63 was shown experimentally to form various possible interactions, such as the triplet with G36–C67 observed in the crystal structure (28), or with U44–A65 as observed in Exp4 (32). The major groove of the GAUC/GAUC helix may therefore be accessible or not, depending on the interactions formed by U63.

Model E, derived from Exp3, exhibits no triplets, and the resulting extended structure is bent.

Finally, the NMR structure from Pham *et al.* (Exp4) exhibits T2, and therefore falls into the M2 category. It was classified as a subset of M2, indicated in the following as M2*, to account for the formation of triplet U63–U44–A65. Potential implications of the formation of this triplet will be discussed.

All structures can be distinguished using the relative position of U40, as measured by the angle ξ formed between U40, the base-pair A43–U66, and the base-pair C45–G64, as indicated in Figure 2. This angle effectively describes the orientation of the backbone below the GAUC/GAUC repeat, and hence the position of U40, with respect to the orientation of the double-helix of the GAUC/GAUC repeat. The triplet T1 is characterized by a value of ξ close to 30° while that for T2 ξ is 90°. Values of ξ greater than 100° are typical for extended structures. In our analysis, we use ξ as a coordinate to distinguish between the different conformational basins: hence, M1 is associated with ξ ≤ 60°, M2 with 60° ≤ ξ ≤ 100°, and E with ξ ≥ 100°.

### Sequence mutations

The mutant structures were derived from the models M1, M2 and E by changing the sequence for subsequent simulations and analysis. The sequence changes were those tested by Martinez-Zapien *et al.* (28) and by Lebars *et al.* (23) for their ability to bind HEXIM. Following the observation that deletion of U40 and U41 leads to loss of binding (23), the importance of these nucleotides was further probed by three mutations: U40C, U41C and U40C + U41C. For all three a decrease in binding affinity was observed (28), with U40C having a stronger impact than U41C. Furthermore, the GAUC/GAUC motif is important for HEXIM recognition, with a change to GGCC leading to loss of binding (23). A more subtle change from CAUC to GUAC has also been shown to weaken the binding affinity (28). Finally, another set of mutations focuses on the Watson-Crick base pair A39-U68, sitting below the 7SK motif. A single mutation, A39U or A39G, reduces HEXIM binding significantly (28), whereas the restoration of the Watson–Crick base pairings with double mutations A39U–U68A and A39G–U68C results in good binding. All these mutants, namely U40C, U41C, U40C + U41C, GAUC to GGCC (double CG), GAUC to GUAC (double UA), deletion of U63, A39G, A39U, A39U + U68A, have been simulated in this study. A complete list of the simulations performed on the mutated systems is given in Table 1.

**Table 1.** Summary of all the simulations performed for each system, including those already presented in (28)*

Molecule	Path sampling	MD	H-REX
WT Exp1	Yes	-	200 ns
WT Exp2	Yes	-	200 ns
WT Extended	Yes	-	-
WT Exp1(24–87) neutral*	-	200 ns	-
WT Exp1(24–87) charged**	-	200 ns	-
WT Exp2(24–87) neutral*	-	200 ns	-
WT Exp2(24–87) charged**	-	200 ns	-
WT Exp3(24–87)	-	200 ns	-
A39G	Yes	500 ns	-
A39G–U68C	Yes	500 ns	-
A39U	Yes	500 ns	-
A39U–U68A	Yes	500 ns	-
delU63	Yes	-	-
doubleCG	Yes	-	-
doubleUA	Yes	-	-
U40C	Yes	500 ns	100 ns
U40C–U41C	Yes	500 ns	-
U41C	Yes	500 ns	100 ns
M1 + peptide	Yes	500 ns	-
M2 + peptide	Yes	500 ns	-
Extended + peptide	Yes	500 ns	-

Simulations are performed on the RNA structure limited to nucleotides 37–70 unless stated otherwise. **WT Exp2(24–87) charged: simulations performed with protonated C71, C75 and A77.

### Energy landscapes

**Figure 3.**
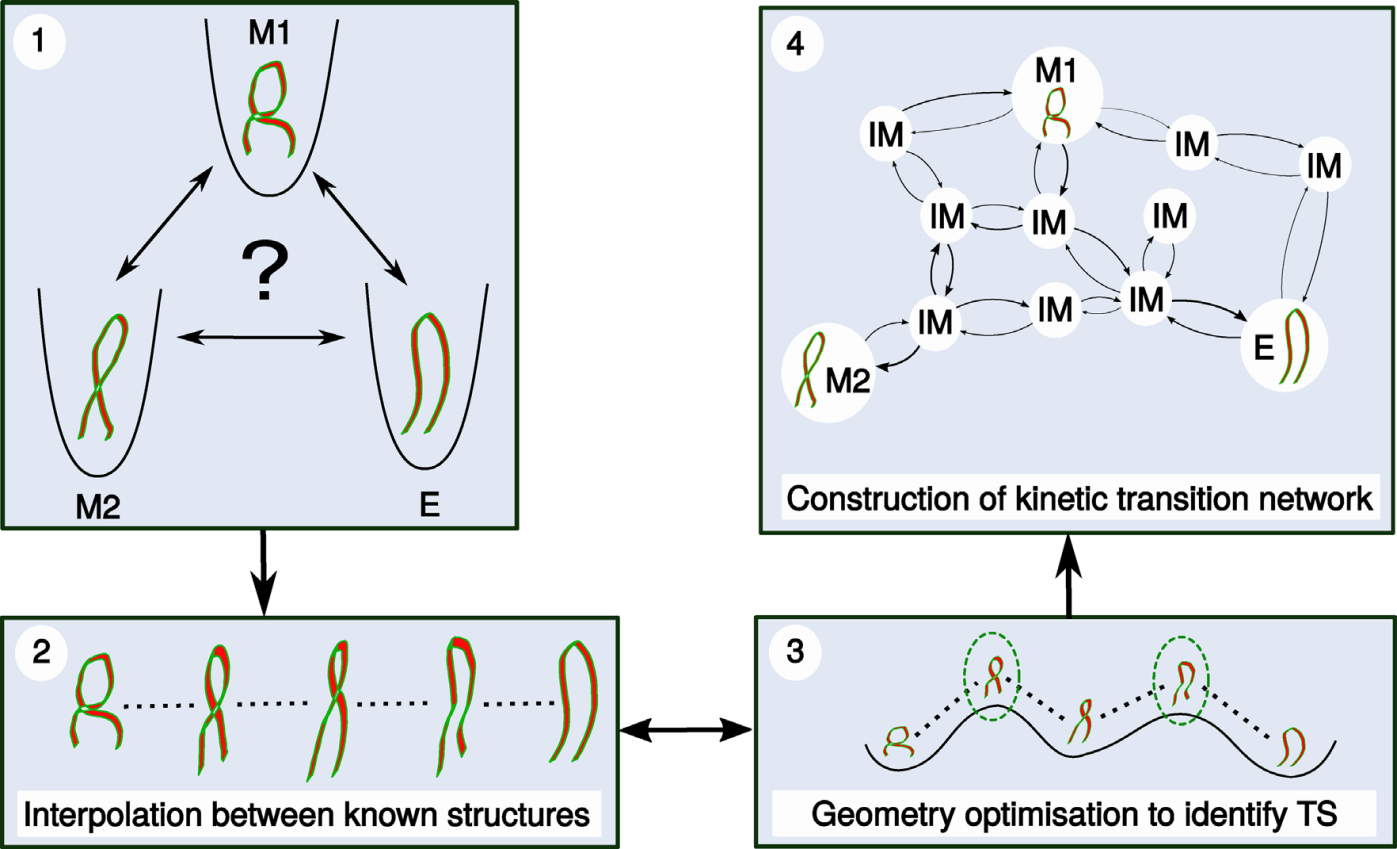
Exploration of the energy landscape to create a kinetic transition network, describing the behaviour of a molecular system. (1) We start with known structures from experimental work (here our models M1, M2 and E), for which we want to understand relative stabilities, populations and the transitions between them. (2) A pair of structures is selected and an interpolation of discrete images constructed. (3) This interpolation band is optimised to produce transition state candidates (dashed green lines). These candidate structures are refined to yield true transition states, which connect two local minima. (4) Full discrete paths (series of minima and transition states) between structures are calculated, and a kinetic transition network can be constructed, including intervening minima (IM). This network describes the energy landscape and allows computation of observable properties and a description of transition mechanisms and stable structures at atomic levels of detail.

The core of this work focuses on the energy landscapes of the hairpin, and exploration of the conformational space of the three models through discrete path sampling (43,44). In this approach we study the ensemble of stable structures (local minima) corresponding to the three experimentally observed conformations to identify their characteristics and relative stabilities. We further locate transition states to identify discrete paths, defined as a series of local minima and the transition states connecting them. Combining these paths into a database produces a kinetic transition network (45,46), which represents the energy landscape. The structural and kinetic data allows us to identify the most stable ensembles of structures. With this approach we can also compute free energies to quantify occupation probabilities, along with transition mechanisms and rate constants. The procedure is described in Figure 3, with more details provided in various reviews (41,47) and in the supporting information.

While energy landscapes contain all the necessary information for us to compute thermodynamic, structural and kinetic properties, they are multi-dimensional surfaces that are hard to visualize. An efficient way to represent them is through disconnectivity graphs (48,49), which are tree graphs connecting local minima and a faithful representation of barriers between them. Hence we can characterize families of structures by their specific properties, distinguishing them from configurations belonging to different funnels.

We applied the energy landscape approach for the short model 37–70 with the wild type sequence, and the mutants described above. The starting points for all calculations were based on the three experimental structures, Exp1, Exp2 and E. An atomistic representation of the systems in implicit solvent is adopted throughout. A similar approach has been used before to study RNA tetraloops with discrete path sampling (50) and transitions between canonical and non-canonical base pairs in DNA (38). Previous studies show that implicit solvent models can yield structures and energies comparable to more expensive explicit solvent simulations (51–54). In particular, the work on RNA tetraloops has been highlighted as an example of implicit solvent simulations that yields good agreement with experiment (54). We also find that binding interactions can be studied in implicit solvent, although explicit solvent yields better numerical agreement with experiment (53). Unfortunately, the use of implicit solvent for nucleic acid simulations has not yet been studied in much detail. The present contribution suggests that implicit solvent can be useful, although careful comparison with other methods, as we have performed in this study, is advisable. More details of the setup are presented in the SI.

### Molecular dynamics simulations

While the energy landscape approach allows for an efficient exploration of the RNA conformational space, the use of implicit solvent could produce unphysical conformations. To strengthen our analysis, we therefore performed all-atom molecular dynamics (MD) simulations in explicit solvent. While they may provide a more accurate description of the RNA–RNA and RNA–solvent interactions, such simulations cannot currently be routinely propagated for more than a few hundred ns, which leads to limited exploration of the conformational space. This issue can be partially addressed using enhanced sampling methods, such as H-REX (see below). A summary of all simulations performed is presented in Table 1.

In our previous study (28), we simulated the wild type HP1-UUCG 24–87 with all neutral bases. After realizing from the crystal structures, that 3 bases in the lower stem are most likely protonated, for the present work we also simulated HP1-UUCG (24–87) with positively charged nucleotides C71, C75 and A77. C71 was also found protonated in Exp4 (32). This preliminary survey was used to assess the possible effect of limiting the rest of our analysis to the shorter model, considering only residues 37–70. Results of these simulations are presented in Supporting Information (section S1, and Supplementary Figures S1 and S2). In the rest of our study the impact of protonation is no longer addressed, since the lower part of the stem is not considered here.

To investigate the structural definition of the 7SK motif, we performed simulation studies on structures with mutations at the 7SK motif itself, and at the U bulge of U40 and U41 and the base-pair A39–U68, that were shown by experiments (23,28) to have an important role on the structuring of the motif.

### Replica exchange MD simulations

Because of the large size of the system and the kind of conformational changes that we want to observe, we also performed Hamiltonian Replica Exchange (H-REX), employing the REST2 variant (55–57). The REST2 algorithm can be successfully applied to biomolecules in explicit solvent, which is very challenging using more conventional temperature replica exchange. In the REST2 approach only the Hamiltonian of the biomolecule is rescaled (i.e. its potential energy). Details about this algorithm and its application to biomolecules can be found in the SI and elsewhere (55–57). Briefly, multiple copies (the replicas) of the systems are propagated. For each replica, the RNA potential energy is rescaled according to a factor λ ranging from 0 to 1. For λ = 1 the system behaves according to the normal interaction strength, while for λ < 1 interactions become weaker and the molecule can easily deform from its original conformation and explore other structures. At fixed time intervals the replicas may exchange, following detailed balance. In this approach, the sampling of the reference replica at λ = 1 benefits from the faster exploration of the conformational ensemble by the replicas with lower λ < 1, while it still evolves with unperturbed interactions.

Replica exchange simulations are not able to sample the energy landscape as efficiently as discrete path sampling, at least for such a large biomolecule. However, the fact that one can use explicit solvent and also obtain some measure of entropic effects is informative. Our results suggest that both strategies lead to conceptually similar conclusions, as detailed in the Results section.

In our study, we analyse H-REX simulations with the same tools used to characterize the states identified from the energy landscape, and compare them to the funnels from path sampling to test whether explicit solvent simulations recover the same structures found in implicit solvent. Finding the same structures when water and ions are introduced back into the system provides confidence in the results for implicit solvent. This approach is similar to what is commonly done in coarse-grained modelling, where full atomistic simulations are used to characterize the viability and stability of configurations proposed by coarse-grained simulations (54,58–60).

For the wild type, we ran H-REX using the two experimental structures Exp1 and Exp2, truncated to nucleotides 37–70, as starting configurations to study the behaviour of M1 and M2. A statistical analysis of the H-REX simulations is presented in SI (Supplementary Tables S2 and S3, Supplementary Figures S10 and S11). Results reported here refers to the second half of the trajectories, where simulations from Exp1 and from Exp2 exhibit converged behaviour (on the simulation timescale).

For mutants, we ran H-REX for U40C and U41C, because of the key role of these two residues in the 7SK motif.

### Peptide binding

The binding with the arginine rich motif (ARM) of sequence GKKKHRRRPSKKKRHWK was probed using basin-hopping global optimization (61–63) and molecular dynamics simulations in explicit solvent. This sequence corresponds to amino acids 149–165 from the human protein HEXIM1. Low energy structures from the energy landscape of the wild type were used, and the peptide was placed into these configurations using PyMOL (64) and then docked using global optimization. We then used MD simulations in explicit solvent to further test the binding between ARM and HP1 for M1, M2 and E. Details of these simulations are provided in the supporting information. We analysed the trajectories with respect to structural variety, i.e. whether structures located were stable or not, looking at the entire system as well as the RNA and peptide individually. We further computed the interaction energy between the peptide and the RNA and analysed the interactions between the two components.

## RESULTS

### The energy landscape for the native sequence

#### Relative populations

**Figure 4.**
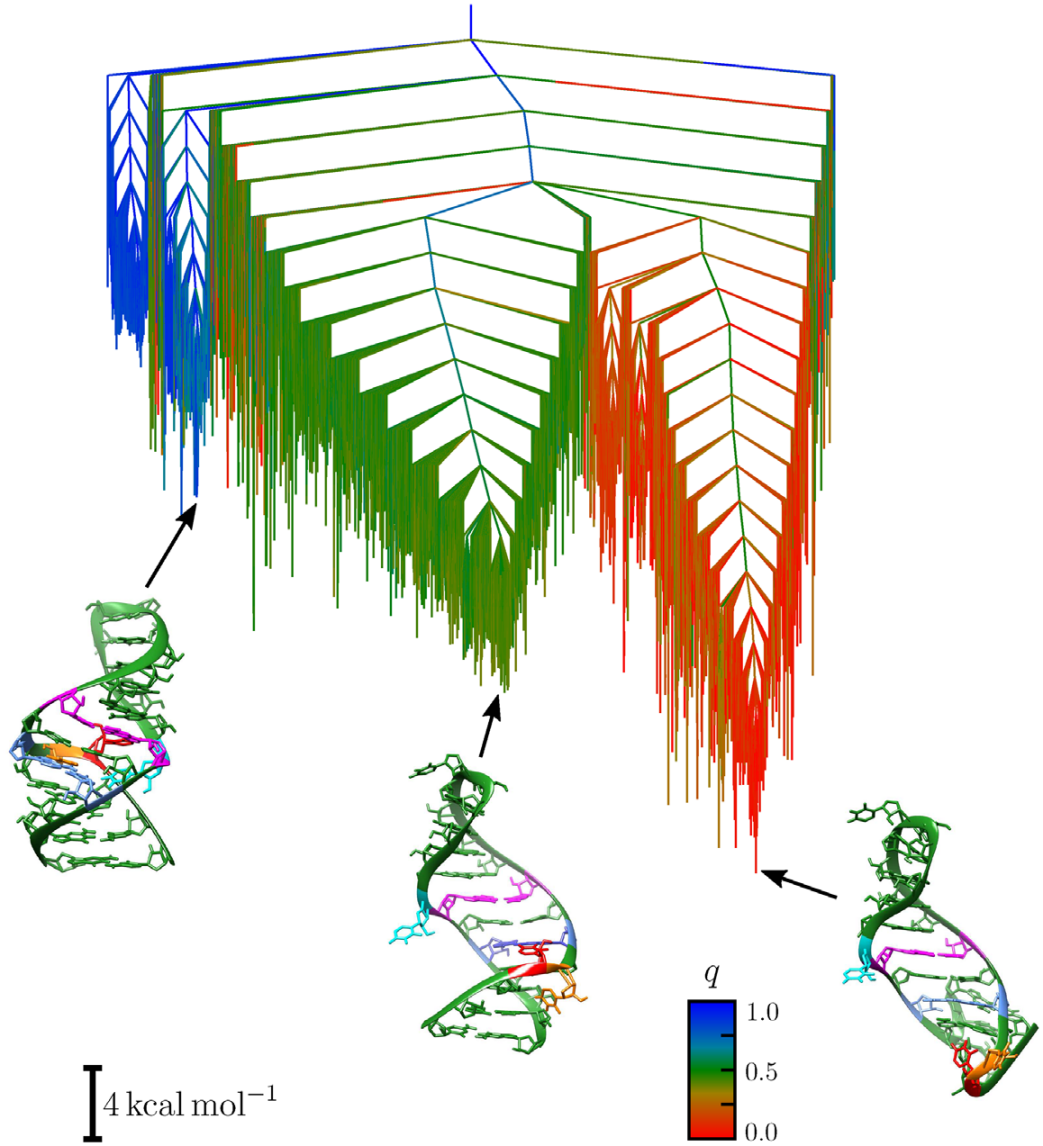
Disconnectivity graph for the potential energy landscape of the native sequence of HP1. The three experimental structures are located in distinct funnels, with the E configuration (red) lowest in energy, and the M1 configuration highest in energy (blue). The 3D structural representations were created using the UCSF Chimera package (33).

The potential energy landscape for the native sequence is displayed in a disconnectivity graph in Figure 4. The landscape is partitioned into three distinct regions, which correspond to the three models derived from experimentally observed configurations: M1 (blue), M2 (green) and E (red). The lowest energy configurations are extended structures (type E), followed by M2, while the M1 state has a much higher energy. The bottom of the funnel containing the E configuration is approximately 5 kcal/mol lower in free energy than the bottom of the M2 funnel, with the M1 funnel 14 kcal/mol higher in free energy at 298 K. Because of these energy differences it is likely that M2 and E could co-exist at physiological temperatures, but not M1. The relative occupation probabilities derived for the E and M2 states indicate that both should be observable at equilibrium, with the E configuration more heavily populated. However, the occupation probability of E type structures is probably overestimated. Indeed, the shortened model allows the closing base pairs of the stem to bend back upwards in an unphysical fashion. This distortion is observed for a subset of the structures in the E configuration, resulting in an artificial stabilization of some structures. MD simulations suggest that extended configurations are possibly less relevant. Indeed they are not found in the all-atom trajectories: the root-mean-square-deviation (RMSD) of the configurations from MD initiated from E is above 6 Å with fluctuations of ±1 Å, indicating significant differences between the structures sampled by the trajectory and the initial E configuration. These differences also include rearrangements in the secondary structure.

H-REX simulations support the observations from path sampling that M2 is more stable than M1, with a strong prevalence of M2 models in simulations originating both from the Exp1 and from Exp2. An estimate of the family content of the two simulations, based on the value of ξ, gives 6% M1, 68% M2 and 27% E for Exp1, and 2% M1, 64% M2 and 36% E for Exp2. These percentages can be compared to the family content computed for path sampling. For this purpose, we consider subsets of the minima database with an energetic cutoff of 15 kcal/mol from the bottom of the M1 and M2 funnels, corresponding to transition times faster than the millisecond scale (65). The content of the M1 basins is 2% M1, 69% M2, 29% E and for M2 it is 0% M1, 95% M2 and 5% E. Structures found in the M1 and in the M2 funnels from path sampling have an average root-mean-square deviation (RMSD) from structures of the H-REX trajectories of 2.2 Å. Overall, these results suggest that (i) after an initial transition toward a more favourable conformations, H-REX simulations starting from Exp1 and Exp2 converge to similar populations on our simulation timescale, (ii) the results of H-REX are in excellent agreement, both in terms of populations and geometries, with discrete path sampling. These results suggest that despite the inherent approximations of both approaches, they lead to very similar results.

#### Characterization of the structural ensembles

Analysing the three structural ensembles on the landscape, we can classify them into the three models, which correspond to clearly distinct funnels. The triplet formation we use as a classification criterion is consistently observed, even for higher energy structures, although some distortions are possible, in particular in the configurations classified as M1. Some variability is present with respect to the T1 triplet. U40 slightly twists upwards out of the plane, and hence space opens up underneath, and U63 can sandwich between U41 and U40. In some higher energy structures U40 moves even higher and starts to interact with the next base pair, G46-C62. The average number of hydrogen bonds for U40 and U41 is between 2 and 3 for each nucleotide, emphasizing the compactness.

The configurations classified as M2 support the T2 triplet with little distortion, along with a subset of M2* configurations at higher energies. U40 is consistently involved in triplet formation, so that the nucleotide is fixed in place. Finally, in the E configurations we do not observe any triplet formation, and there are no interactions formed by U40 or U41. The distance between U63 and U40 is significantly increased, and U40 can move freely.

In all-atom H-REX simulations, the structures tend to adopt a less compact conformation than in experiment. The radius of gyration evolves from 12.5 and 12.9 Å for Exp1 and Exp2 to values fluctuating around 13.5 Å for both (see Figure S5). This result is not surprising, since the configurations used to initiate the simulation come from crystals, while MD is intended to reproduce solution conditions at finite temperature. The evolution of ξ exhibits a drift of Exp1 toward values typical of M2, while Exp2 remains stable with fluctuations of ξ around its initial value. Both simulations also explore extended configurations (E). Indeed, in the simulation starting from Exp1, T1 is rapidly lost, with U40 positioned at the level of A43, at times engaging in the triplet T2. T3 is also rapidly lost and never reforms. In the simulation starting from Exp2, T2 breaks and reforms, with U40 detaching from A43.

Regarding U63, in the M1 structures, U63 is in the centre of the fold and it cannot move out without disruption of both triplets. In the M2 structures from path sampling, we see a preference for U63 to point away from the fold, opening the major groove and exposing the GAUC/GAUC motif. We do not see the triplet of M2* (32) as a stable structure, but we observe M2* configurations higher in energy. In the MD simulations U63 fluctuates between in and out positions. When pointing inside the major groove it can stack with U40, especially if this base is not already involved in T2 and can move into the groove approaching U63 more easily.

**Figure 5.**
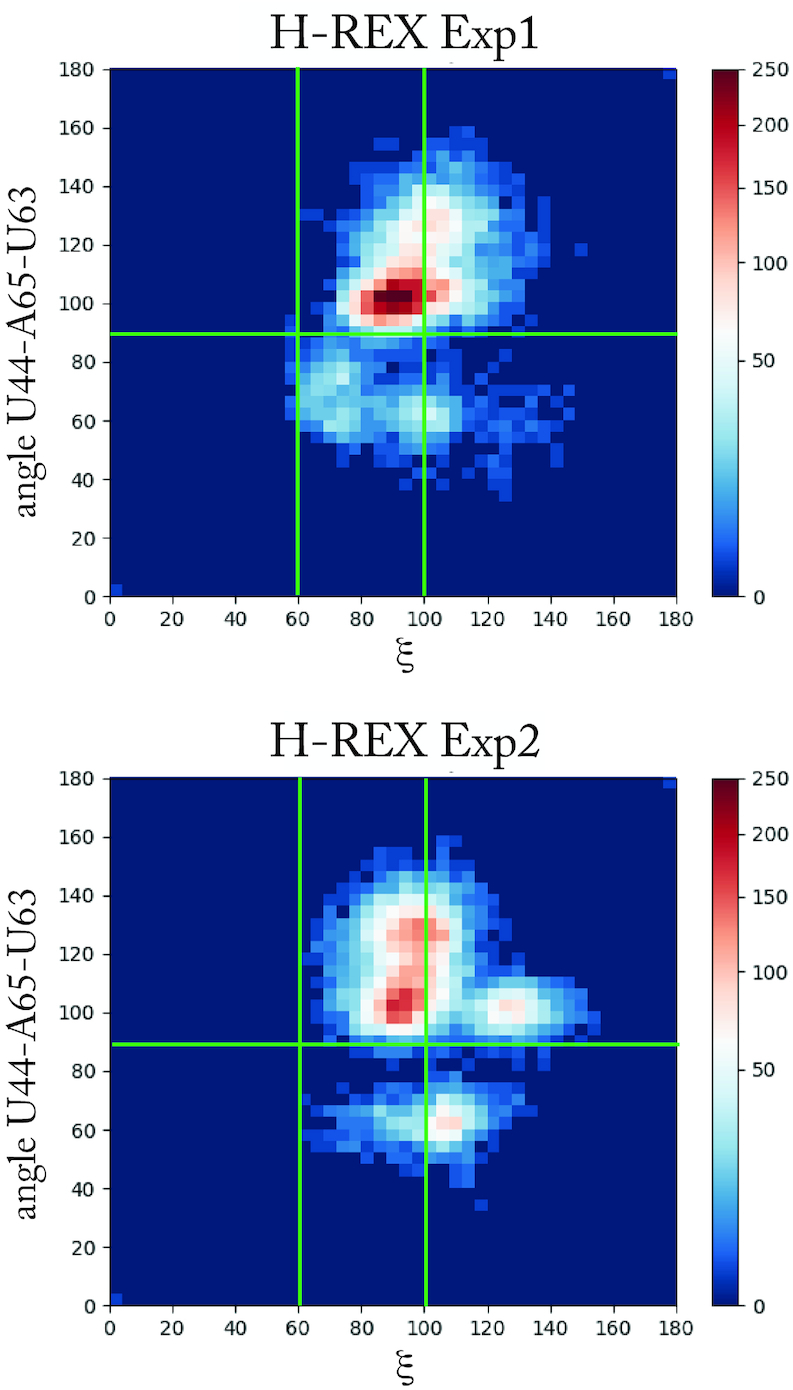
Projection of the structures sampled by H-REX onto the variable ξ, indicating the family to which the structure belongs, and the angle U44–A65–U33, indicating the inward or outward position of U63. A similar plot for the path sampling results is provided in Supplementary Figure S3.

The diversity of conformations explored by the simulations can be visualized by projecting the population on namely two axes, ξ, indicating the family, and the angle U44–A65–U63, indicating whether U63 points inward or outward. Figure 5 shows such a heat-plot for the two H-REX simulations, indicating the structural content of each ensemble. For comparison, a similar plot is given in Supplementary Figure S3 for the structures from path sampling.

#### Transitions between structures

**Figure 6.**
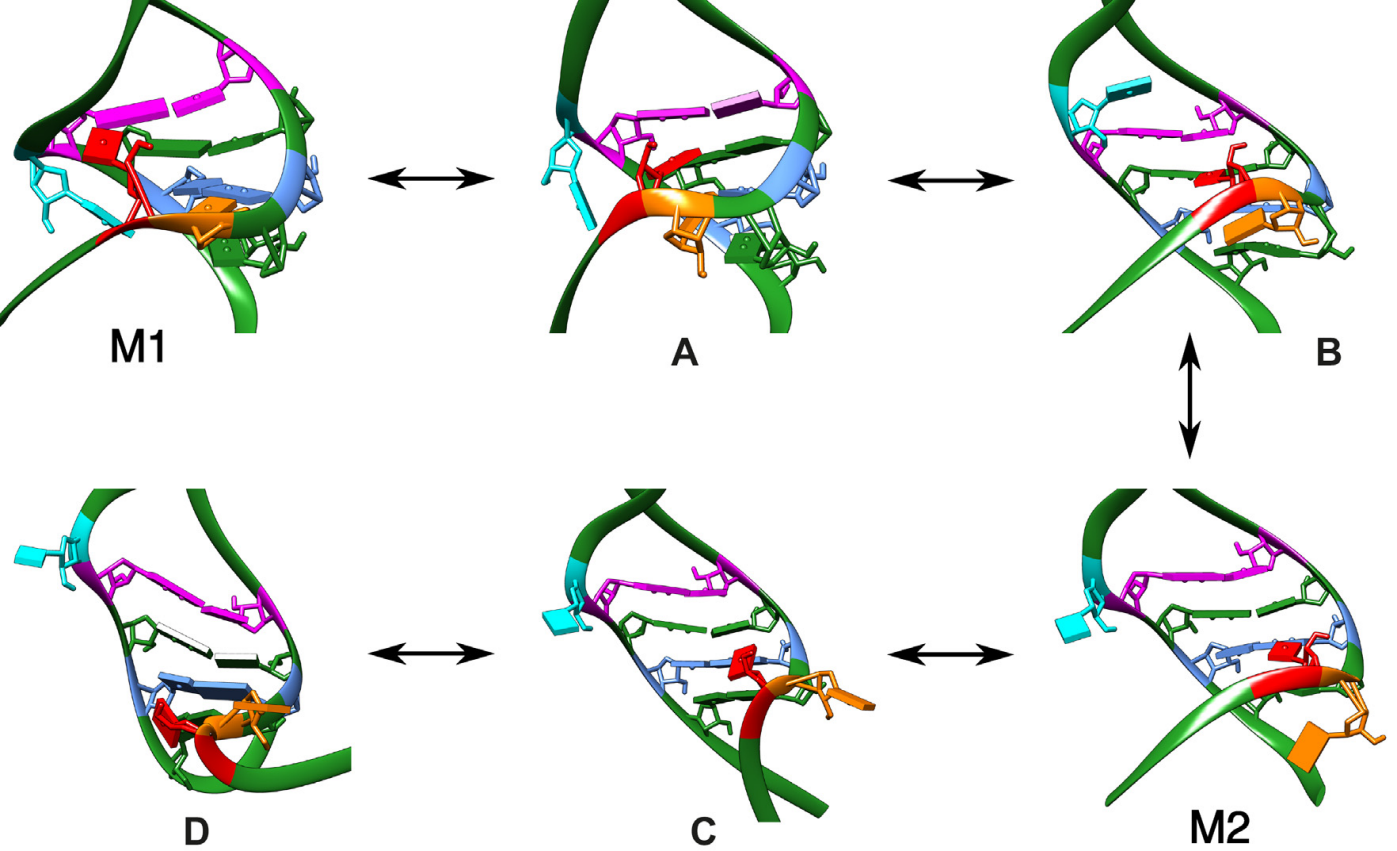
Structures illustrating the key intermediates in the transition from M1 to M2 and M2 to extended structures. In intermediate (a) U40 (red) is no longer found forming T1, forcing U41 downwards and out of T3. U40 can slip further down the strand to form T2 (intermediate b). This structure relaxes to M2. Movement in the lower part of the structure destabilizes T2 (intermediate c), which can lead to the loss of the triplet and formation of extended structures (d).

The transition mechanisms between the three ensembles can also be analysed from the energy landscape. For the transition from M2 to E configurations, the T2 triplet is lost by motion of U40, and subsequently the backbone rearranges to release strain due to the bulging of the unpaired uracils. The process has an estimated exchange rate of about 10^−2^ s^−1^ at room temperature. The transition from M1 to M2 includes an intermediate structure. First U40 moves from C45–G64 to form a triplet with U44–A65, which leads to strain on the T3 triplet and U63. This strain is released by slipping of U63 out of the major groove, extending the structure, to form a stable intermediate. This structure exhibits distortion of the base pairs in the GAUC/GAUC motif, as they are bent out of plane. U40 then moves to form T2. While the upper part of the hairpin is now in a stable configuration, the backbone around U41 is twisted, and the final relaxation to a M2 structure requires therefore backbone motion. This transition has much higher associated energy barriers, as the structure needs to distort in the process, with a calculated exchange rate between 10^−4^ s^−1^ and 10^−3^ s^−1^ at room temperature.The rate constants for the forward and backward process vary by about ten orders of magnitude, meaning the M1 to M2 transition will be very fast, compared to the reverse transition. As the transition from M1 to E and vice versa involves the motion of U40 down or up along the structure, these transitions follow pathways with M2 configurations as intermediates. Characteristic structures along the transition pathways are shown in Figure 6.

### Binding of ARM

In the analysis of the MD simulations of M2, M1 and E with the ARM peptide, we observe drastic differences, both in terms of the interaction energy, and in the details of the specific interactions formed.

In the M2 configuration the major groove is open and the peptide sits stably inside. U40 and U63 are accessible to the peptide, and both form interactions. In the M1 configuration the major groove is obstructed by the triplet T1 and the peptide cannot penetrate. As a consequence it explores the external surface of the RNA without finding a stable conformation. In the extended configuration E, the major groove is open, but U40 and U63 are distant and do not form stable interactions with the peptide, which explores various possible conformations, again not finding a favourable position.

**Figure 7.**
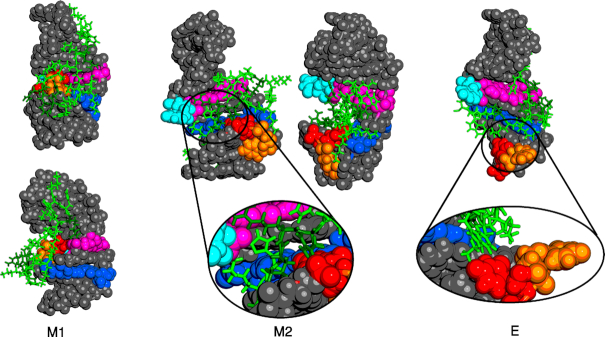
Lowest energy structures located for the binding of ARM to the shortened RNA 7SK hairpin model. Left: For the M1 configuration the peptide (green) is only on the surface of the RNA, and due to the closed major groove, no favourable interactions can form. Centre: In the M2 configuration the peptide can access the major groove and form interactions with the nucleotides of the hairpin, in particular with nucleotides of the GAUC/GAUC motif. The Arg residues (dark green) are deep in the opened groove and interact strongly with U63 (cyan) and U40 (red). The proline in the middle of ARM is also deeply buried, giving the peptide the flexibility to interact simultaneously with the GAUC/GAUC, U40 and U63. Right: In the extended configuration the 7SK motif is exposed, but the distance between U63 and U40 is increased, preventing strong interactions of the peptide with all three regions of the RNA at the same time, lowering the binding energy, and potentially the binding specificity.

Because of the charged nature of both molecules, the dominant interaction energy is Coulombic. The M2 configuration is by far the lowest in energy, indicating a stable, bound configuration, which is shown in Figure 7. The ARM motif has a number of charged residues, which sit deep inside the open major groove. Not only does this arrangement lead to enthalpically favourable contributions, but solvent molecules will be excluded from the groove and from the peptide, increasing the entropic driving force for this binding process. This observation is further supported by the fact that the MD simulations exhibit very little motion for the M2-peptide system. The RMSD in the MD simulations for M2 fluctuates between 1 and 2 Å. In contrast, for the E and M1 configurations no stable bound complex was observed. For E we observe RMSD fluctuations larger than 8 Å, and the Coulombic interaction energy is weakened by around 35%. In the extended configuration, the absence of a direct interaction between U40 and U63 with the peptide reduces the overall interaction energy by ∼15%.

**Figure 8.**
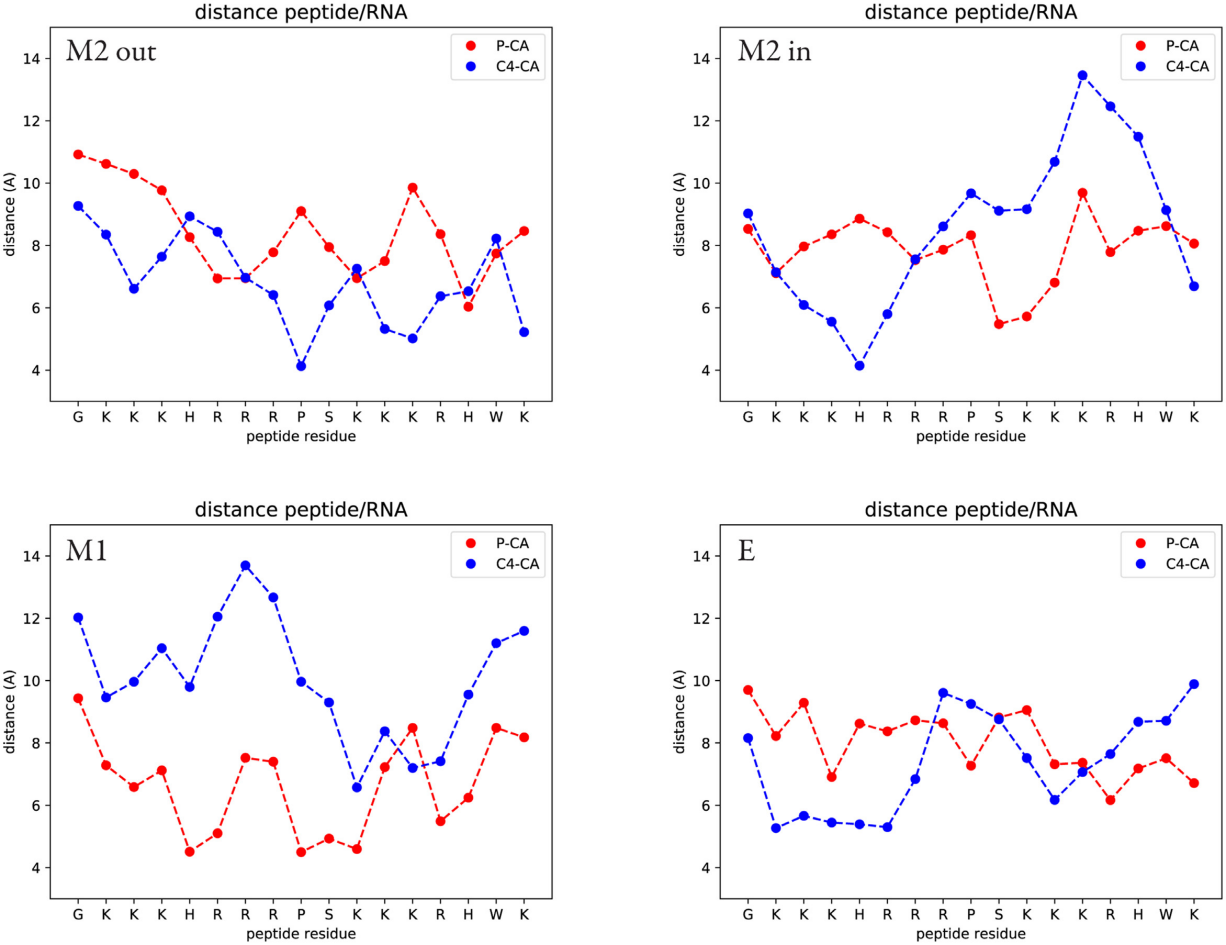
Average distances of the residues of the ARM peptide from the RNA as measured from the CA atom of the amino acid to either the closest phosphate atom (P), indicating the distance of the amino acid to the RNA backbone, or the closest C4 atom, indicating the distance of the amino acid to the RNA bases. Averages are performed on the MD trajectories for the 4 systems.

To investigate the role of U41, which has been highlighted as potentially interesting for the binding process (28), we tested two distinct arrangements of U41 within the M2 configuration: U41 pointing away from the RNA strand (‘M2 out’), and U41 pointing inwards and sitting below T2 (‘M2 in’). We have further analysed the MD trajectory to understand the details of the interactions between the peptide and the RNA for the four configurations: ‘M2 out’, ‘M2 in’, M1 and E, looking in particular at the penetration of the peptide into the RNA groove and on the hydrogen bond network formed between the two partners. Figure 8 shows the average distance between the residues of the ARM motif and the RNA for the four systems. We measure the distance between the CA atom of the amino acid to the closest phosphate of the RNA and the distance between CA and the closest C4 atom on the RNA bases. The first gives a measure of how close the amino acid is to the backbone, while the second gives the information of how much the peptide enters the groove. For ‘M2 out’ we observe a regular pattern in which the charged amino acids clearly interact with the RNA backbone all through the peptide chain, while the proline is deeply buried inside the groove, close to the bases. The peptide penetrates the groove in all its length. On the contrary, for the other three structures the peptide sits close to the RNA backbone, but remains far from the bases. Therefore the peptide is close to the RNA, minimizing the electrostatic interactions, but does not enter into the groove.

**Figure 9.**
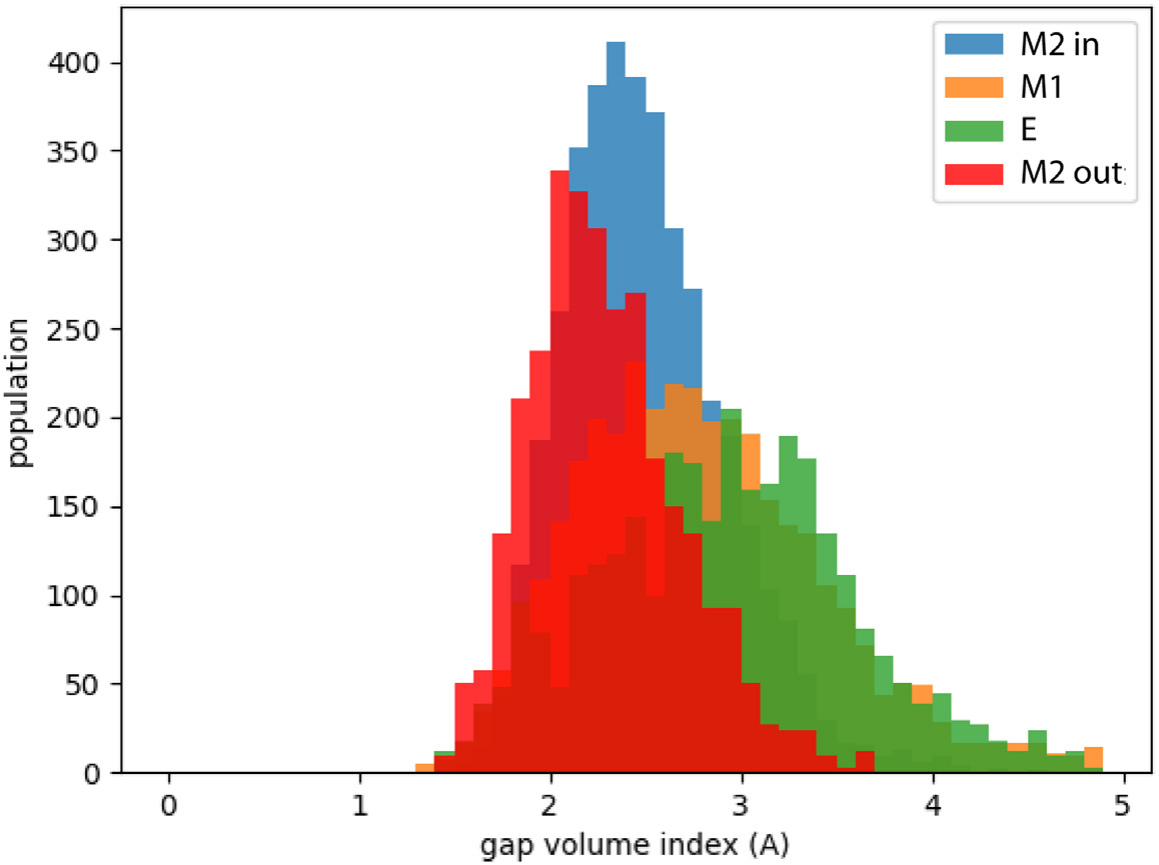
Distributions of the gap index (*g*) for the four systems. The computed average values are: <*g*_*M*2*out*_ > =2.3 ± 0.4; <*g*_*M*2*in*_ > =2.4 ± 0.4; <*g*_*M*1_ > =2.8 ± 0.7; <*g*_*kink*_> =2.9 ± 0.7.

Another measure of this behaviour is given by the value of the gap index (66), which is the ratio of the volume of the gap between two structures and the interface surface. Small values indicate that one partner is deeply buried in the other. The distributions of the gap index from the trajectories, shown in Figure 9, highlight once more that for ‘M2 out’ the peptide is in closer contact with the RNA than for all other structures.

Last, we looked at the hydrogen bonds formed between the peptide and the RNA and their persistence during the simulation (Supplementary Figure S4). ‘M2 out’ forms stable contacts with the GAUC/GAUC motif, branching to the opposite strands of the RNA. A similar pattern is observed for ‘M2 in’, but with lower persistence of the bonds. M1 and E exhibit different interaction patterns, not involving the 7SK motif and with generally low persistence. We also observe that in ‘M2 out’ and in ‘M2 in’ U40 is involved in hydrogen bonds with the peptide, as opposed to M1 and E.

All these observations suggest that the M2 configuration is the structure that actually binds the ARM motif.

### The effects of mutations

**Figure 10.**
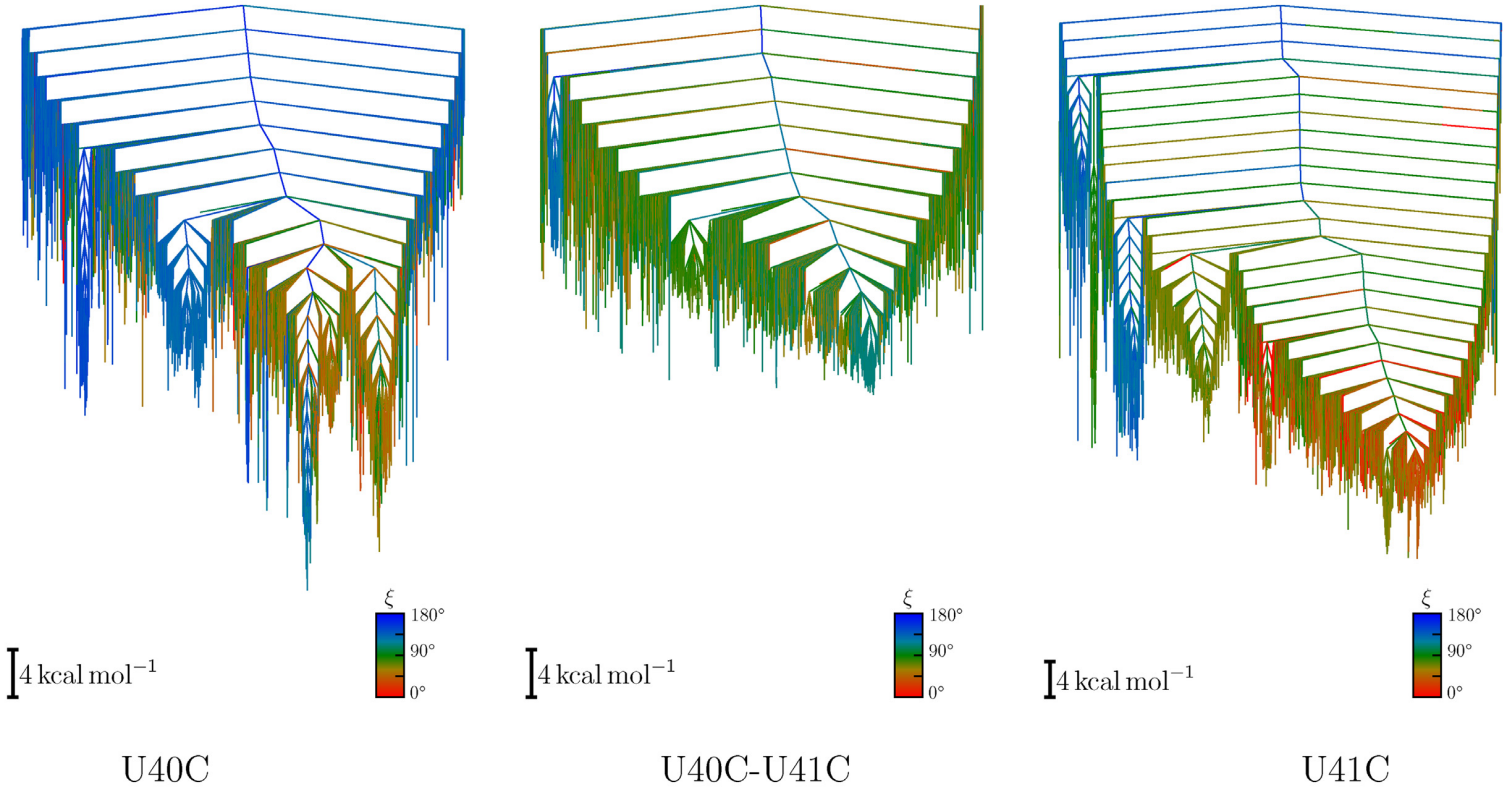
Disconnectivity graphs for the U40C, U40C–U41C and U41C mutants compare the altered landscapes upon mutation. For the U40C mutant the lowest funnel consists of M1 structures with the second-lowest funnel being mainly extended configurations. Very few M2 configurations are found (dark green), and a similar picture emerges for U40C + U41C. For U41C, the lowest funnel is broad, and contains a mixture of E and M2 structures, but some compact configurations are still found at low energies.

**Figure 11.**
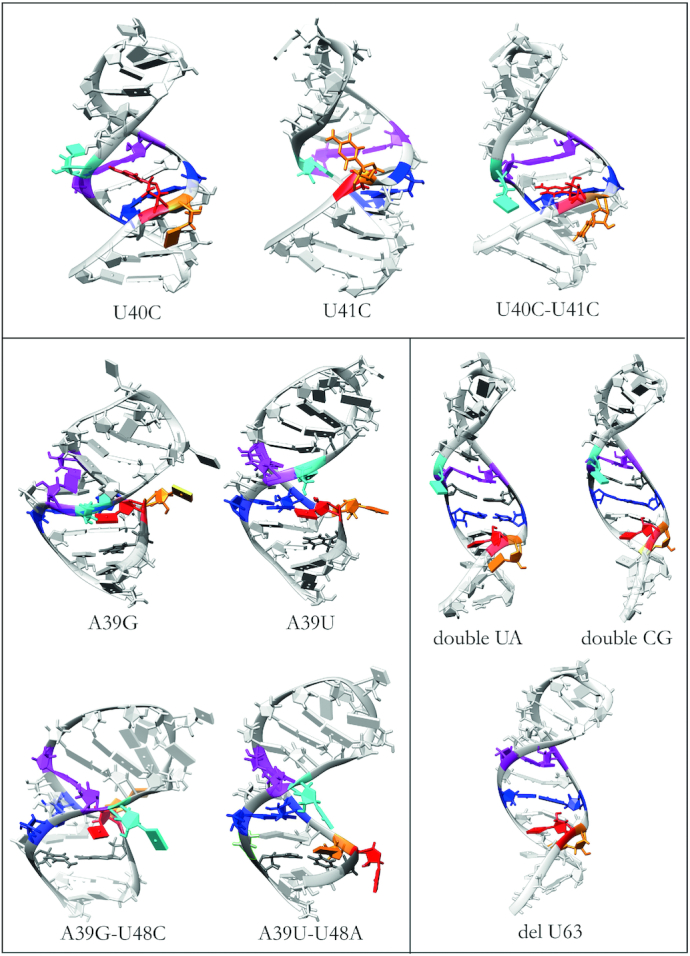
Representative conformations for the most populated structures of each mutation following the colour code: U40 (orange), U41 (red), A43 and U65 (blue), C45 and G64 (magenta), U63 (cyan).

From both path sampling and MD simulations, we can look at the effect of mutations on M2, M1 and E and characterize the most populated structures. Here, we report the general features for the behaviour of each mutant, giving a qualitative analysis. The key features were extracted from a systematic quantitative analysis, for which details are provided in supplementary information. In Supplementary Table S1 we report energies, average hydrogen bonds, family content, U63 orientation for each minimum identified by path sampling. In Supplementary Figures S6 to S9 we report disconnectivity graphs and heat plots for each mutant, with one set of disconnectivity graphs also provided as an illustrative example in Figure 10. Example structures for all mutants are shown in Figure 11.

#### Mutations of U40 and U41

For the U40C mutant, a key feature is the altered pattern of interactions of U63. While in the wild type we observed in and out configurations, in this mutant U63 is either pointing inwards or interacting with nucleotides in the opposing strand. This limited set of configurations closes the fold, making the GAUC/GAUC motif inaccessible from the major groove side. Furthermore, very few structures exhibit a M2 configuration, and instead many kinked, low energy structures are observed, exhibiting C40–U68 interactions. As a result, C40 is not available for binding, and blocks access to the major groove.

For the double mutation U40C + U41C the structural ensemble observed is compact, with a large number of hydrogen-bonding interactions for both C40 and C41. Indeed we observe an average of 3.3 hydrogen bonds for the C40 residue and 2.6 for C41, instead of 2 and 0, respectively, for Exp2. Consequently, the structure does not expose the major groove of the GAUC/GAUC motif.

In contrast, the U41C mutant exhibits the largest variety of structures among this set of mutants. Again we observe extended configurations with U40-U68 interactions, but there are also low-energy structures in the M2 configuration with the T2 triplet formed and U63 pointing out. The three disconnectivity graphs for these mutants are shown in Figure 10.

Full atomistic simulations, both serial and replica exchange, lead to structures very similar to those found by path sampling, with RMSD values as low as 1 to 3 Å for the low energy structures, indicating an important structural similarity. When a cytosine is substituted in position 40 the triplet T2 becomes less stable, and the base C40 is free to explore alternative conformations. As also observed in implicit solvent, these structures often involve hydrogen bonds with other bases inside the groove or with phosphate groups across the groove. When a cytosine is substituted in position 41 the triplet T2 remains stable, however C41 can move towards the inside of the groove, forming hydrogen bonds in the same way as for the mutation at position 40. The most remarkable effect of these mutations therefore appears to be blocking of the major groove by the mutated cytosine, which can form a tight network of hydrogen bonded interactions inside the groove.

#### Mutations of GUAC/GUAC and deletion of U63

Another set of mutations addresses the GAUC/GAUC motif and U63 that regulate the accessibility and the structure of the binding motif. The three sequence changes that fall into this category are two mutations of the GAUC/GAUC motif, namely GAUC to GUAC (doubleUA) and GAUC to GGCC (doubleCG), and the deletion of U63 (delU63).

The deletion of U63 makes the pairing of U40 with U68 more likely, as it moves the strand up towards the hairpin, and consequently the most favourable structure observed is an extended configuration, with U40 forming a base pair. However, there are accessible low energy structures that exhibit the M2 configuration, still allowing for binding.

In contrast, for the doubleUA and doubleCG mutants the lowest energy structures in both cases have extended configurations, with the U40-U68 base pair formed. While some M2 configurations exist at higher energy, most high energy structures are kinked configurations, exhibiting T3 triplets blocking the major groove of the GAUC/GAUC motif, or M1 configurations.

#### Mutations of A39–U68

The final set of mutations examines the importance of the base pair A39–U68, which is next to the unpaired nucleotide U40. In experimental structures Exp 1–3 the canonical Watson-Crick base pair is formed, in contrast to Exp4, where base pairing between A39 and U68 is detected upon binding to Tat but not for the RNA alone. Stabilization of this base-pair upon peptide binding was also observed in earlier studies with the HEXIM peptide by Lebars *et al.* (23).

In the A39U mutant, the structural ensemble shifts towards extended structures, in some of which U40–U68 interactions form. In other structures the destabilization of the base pair leads to adjustments in the backbone and results in kinking, either pulling U63 closer to U40, leading to interactions that close the groove and shielded the GAUC/GAUC motif, or allowing stacking interactions of U63 and destabilizing the T2 triplet. Similar results are observed in fully atomistic MD with the formation of a mostly stable U40–U68 pair and with U63 points inward, sitting next to G34. T2 is formed most of the time, but exhibits some fluctuations.

For the A39G mutant, many of the low energy structures are very compact, and while they exhibit the T2 triplet, U63 also sits in the fold, closing the groove. There are low energy M2 configurations, where the fold is still relatively compact due to disorder in the G39–U68 base pair. Alternatively, U63 is involved in stacking interactions. Interestingly, we do not observe any states with a U40–U68 base pairing. In fully atomistic MD we observe a stable G39–U68 pair and U63 pointing inward, blocking the groove, either sitting next to G34, or reaching across the groove.

For the two double mutations, A39G + U68C and A39U + U68A, the energy landscapes exhibit low energy structural ensembles for the M2 configuration, with U63 either pointing out, or only weakly bound, with enough flexibility to move out, without encountering a significant energy barrier. A number of other structures are more compact than M2, with additional interactions of U63 and U40, but nearly no extended structures have been located, likely due to the stable 39-68 base pairing.

For A39G + U68C, atomistic MD simulations suggest a very stable G–C canonical pair, U63 mainly in the open conformation, and U40 engaging in the T2 triplet. In contrast, for A39U+U68A, MD simulations suggest a more unstable binding site with disruption of the T2 triplet in favour of the formation of transient base pairs between U40 or U41 with A68, on the opposite strand. As a result the three consecutive nucleotides U39, U40 and U41 swap positions frequently and stacking between U40 and U63 is observed.

## DISCUSSION

### Three distinct structures

The first result of our work is a detailed comparison of the three experimental structures that we also detect in our simulations as three distinct families. The energy barriers we compute explain why different experiments may detect only one or two of the structures, depending on the conditions.

From our simulations we observe that M2 is the structure with the lowest energy and it has a high probability of being populated, as shown both by the energy landscape analysis and by MD trajectories. We observe fluctuations in the position of U63, and detect M2* as a high-energy subset of the larger M2 family.

The M1 configuration is compact and exhibits two triplets and a plethora of interactions between U40, U41, U63 and the GAUC/GAUC motif. This tight packing leads to inaccessibility of U40 and the major groove of the GAUC/GAUC motif, and U63 points consistently inward. For the extended configurations, the major groove of the GAUC/GAUC motif and U63 are solvent exposed. The extension puts strain on the A39–U68 Watson–Crick base pair, as U40 and U41 are moving freely. This strain can be released if the U40–U68 base pair is formed, in which case stacking interactions are observed for both U40 with A39 and U41 with U40.

The transitions between the three distinct structural families are correlated to the motion of U40 up and down along the base pairs in the GAUC/GAUC motif. As shown in Figure 6, the transition between M1 and E occurs via M2.

### HEXIM binding: the key role of U63 and U40

From our simulations we can now relate specific structural features of the experimental structures and of the mutants to the propensity to bind to a ARM peptide at the 7SK binding site and help elucidate the experimental binding affinities observed in (28). A summary of the main results from all simulations is presented in Table 2 where we also report the measured binding affinities.

**Table 2.** Summary of measured binding of 7SK with HEXIM at different peptide concentrations measured as percentage of complex formation (28), and of the observed behaviour from simulations for wild type and mutants

Molecule	Binding at 0.3 μM	Binding at 0.8 μM	Observed behaviour in simulations
WT	80%	90%	Mainly M2, U63 outward
A39G–U68C	80%	90%	Mainly M2, flexible U63.
A39U–U68A	60%	95%	Mainly M2, flexible U63.
delU63	65%	80%	M2 and extended, some U40–U68
U41C	50%	80%	Few M2, some structures with U63 outward
A39G	40%	80%	Mainly M2, U63 inward
doubleCG	30%	70%	Mainly extended, U40–U68 pair, few M2
doubleUA	30%	70%	Mainly extended, U40–U68 pair, few M2
A39U	30%	60%	Mainly extended, some U40–U68, U63 inward
U40C	20%	65%	Few M2, U63 across the groove
U40C-U41C	15%	45%	Few M2, C40 and C41 unavailable

Quantitative details of the simulations for mutants can be found in Supplementary Table S1.

A hypothesis about the binding for HEXIM and RNA 7SK, which emerged from experiment (28), is that U41 can block the access to the major groove, preventing binding. In our simulations we similarly find that the accessibility of the major groove correlates with the observed binding affinities. However, as U41 may be found in- and outwards in the mutants, we propose that the position of U41 is not the major criterion to explain the outcome of mutations.

Instead, our work points to two features that facilitate binding. First, U63 apparently plays the role of a gatekeeper. If it moves closer into the fold, and potentially forms interactions with U40 or U41, the GAUC/GAUC motif is blocked. If the nucleotide swings out, it opens the fold, providing access to the major groove. Furthermore, our modelling showed that it can interact strongly with the charged amino acids in ARM. Second, is the availability of U40 to form interactions with ARM. The formation of the T2 triplet permits stable interactions, and prevents U40 being involved in base pairing, which would interrupt this process. Both these structural features are contained in the M2 motif, which in this hypothesis would make a strong binding target. The interpretation of the mutational assay can be consistently achieved with this proposed binding mechanism.

High binding affinities correlate with the presence of M2 structures, and the accessibility of the groove, which depend on the position of U63 and on the behaviour of nucleotides 40 and 41. This is the case for the double mutant A39G + U68C, with a mutation stabilizing the binding site by the formation of a strong canonical pair, where the mutant binds to the peptide as effectively as the wild type. In the double mutant A39U + U68A the possible disruption of the binding site and consequent blocking of the major groove agrees with the experimental observation of a diminished binding affinity with respect to the wild type.

All mutations leading to the occupation of the groove or the disruption of the T2 triplet correlate with poor binding affinities. This is the case when we replace one of the uridines at 40 or 41 with a cytosine. For these mutants the groove is no longer accessible because of the network of hydrogen bonds formed by the C inside and across the groove. Of the three mutants at this site, the one having the smallest impact on binding is U41C, which we predict to have a smaller impact on the disruption of T2 and of the groove. The binding affinity of the doubleUA and doubleCG mutants is significantly worse than in the wild type. In our simulations we observe these structures in extended configurations, therefore completely altering the organisation of the binding site, and of T2 in particular. However, some M2 structures are still detected, suggesting a residual binding affinity. Along the same lines, the binding affinities of the single mutants A39U and A39G are reduced with respect to the wild type. For these systems we observe few M2 configurations and the possible presence of U63 inside the groove, blocking access to the binding site.

All of our results point strongly to M2 as the good binding target for HEXIM. The presence of alternative experimental structures, and of E in particular, could be indicative of a regulation mechanism for the binding process, as E is also accessible at physiological temperatures, but it is incapable of binding. Similar behaviour has been found in other biomolecular systems, such as ubiquitin, whose landscape shows a very similar topography to the one observed for RNA 7SK here (67), and in DNA-protein complexes (68).

### HEXIM and Tat: differences in binding

**Figure 12.**
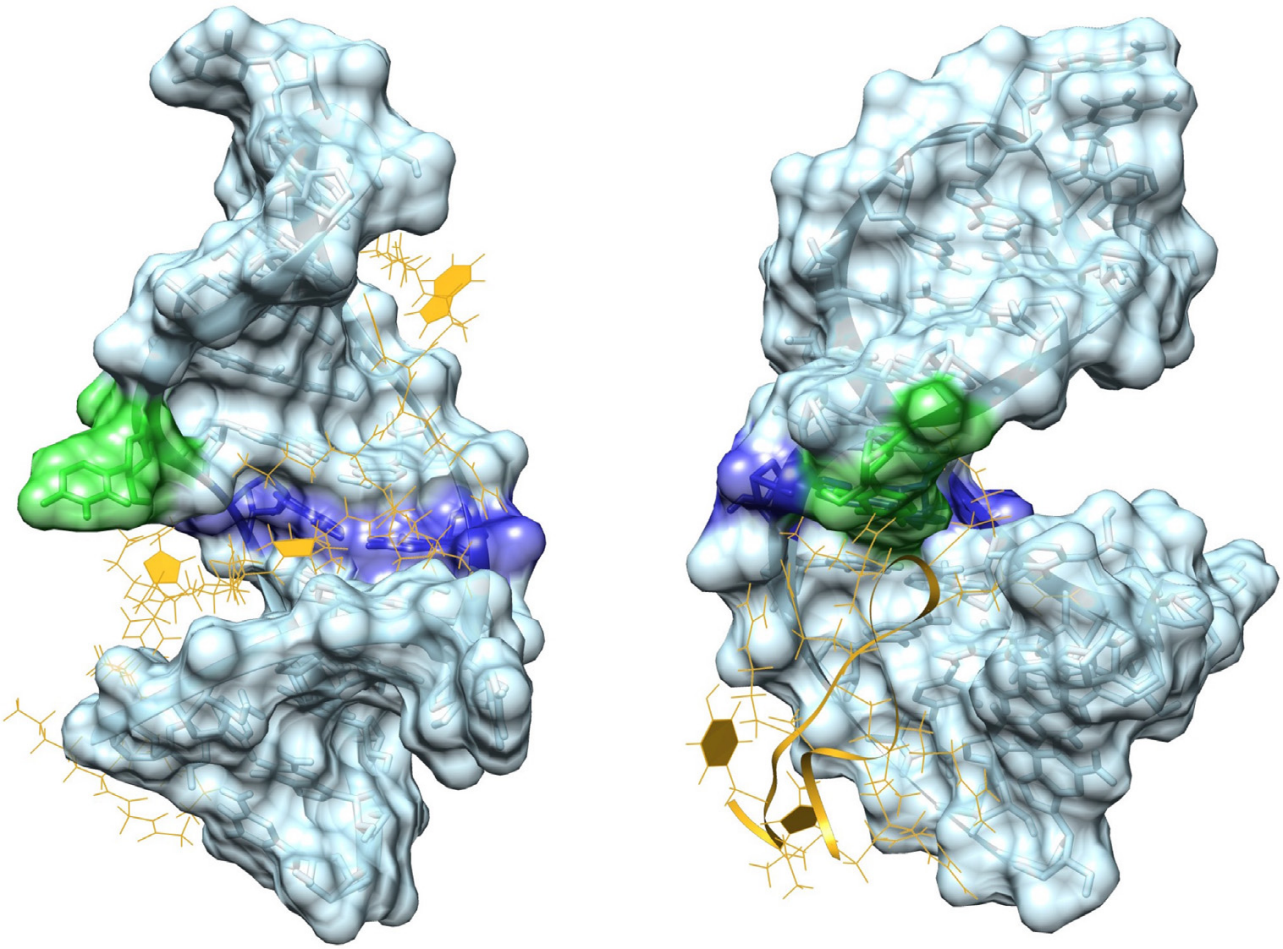
Right: 7SK RNA from the MD simulations with HEXIM peptide. Left: 7SK RNA from the experimental structure of of 7SK with Tat peptide (PDB ID: 6MCF). We highlight the surface of the apical portion of HP1 together with the atomistic structure. U63 is shown in green and the triplet U40–A43–U66 is shown in blue.

Our results exhibit differences compared to the NMR structure reported for Tat binding to RNA 7SK (32). The reported NMR structure exhibits binding to conformation M2*, which is similar to M2, but with a notable change at U63 that forms the triplet U63–U44–A65, absent in M2. Furthermore, for M2 we observe binding in the major groove at the 7SK motif, with key interactions formed between the peptide and U40 and U63. In contrast, the reported NMR structure incorporates the peptide below T2 (Figure 12). Differences in the binding of HEXIM and Tat are expected, as the sequences considered in the two experiments, and in the simulations, are significantly different. In particular, despite both being arginine-rich motifs, characterized by the abundance of positively charged residues, in the central portion of HEXIM we find a proline and a serine instead of the glutamine of Tat. Moreover, in the terminal part the tryptophan of HEXIM is replaced by another glutamine in Tat, replacing an aromatic with a hydophilic amino acid. These differences could be responsible, not only for rearrangements in the local interactions, but also in the global positioning of the peptide around the nucleic acid, potentially leading to differences in the binding process. This point of view would still agree with the observation that Tat and HEXIM utilize the same binding site, but suggests that they might use the binding site differently.

In our simulations we observe fewer interactions than in the NMR structure for Tat, and our proposed binding site is more accessible and exposed than the one found in NMR experiments. The key difference is in the role of U63, which has to be in an open position for the binding of HEXIM to M2, while it is involved in a triplet in the structures with Tat, therefore making the groove access more narrow.

We suggest two reasons for these observations. It could be that the two proteins bind via competitive mechanisms, i.e. that they utilize the same binding site in different ways (32). Alternatively, the two sets of data may correspond to different steps in the binding mechanism. Our simulations potentially describe an earlier stage of the process, where the binding partners form stable interactions, for which the 7SK motif and U40 and U63 are vital, as they are recognized by the binding protein or peptide. Once a stable complex is achieved, it could then rearrange to form the more stable interaction seen in NMR. This idea would also explain how the deep burial of the peptide can be achieved, and how the mutational assay and NMR evidence might be reconciled. In our simulations we would not observe the secondary migration of the RNA, as our model is shortened and the MD time scales simulated are too short to capture this process.

## CONCLUSION

Experimental and simulation results for HP1 highlight the structural plasticity of RNA molecules and suggest the possible functional role of the different structures in the regulation mechanism of binding to a protein partner. Multifunctional proteins have already been associated with multifunnel energy landscapes (35,69), but for non-canonical RNA this connection has not been made before. Our study suggests that the conversion between M2 and E might play a role in the regulation of 7SK activity and sensitive NMR methods, such as CEST (70), might be able to determine the experimental conversion rates between the two structures.

While for some systems there is a large amount of structural data available, extracting a coherent model bridging apparently conflicting data may be challenging, as experiment might not have the temporal and spatial resolution necessary to map out biophysical processes such as RNA – peptide binding in detail. Here, we have shown how simulations may be used to propose an underlying model to reconcile structural data and mutational assays, and describe such processes in atomistic resolution. The energy landscapes presented here for the RNA 7SK hairpin and its mutants, not only provide insight into this complex and important system, but highlight the similarities of theoretical analysis for nucleic acids, in particular of non-coding nucleic acids, and proteins (47).

Possibly the most important aspect of our work is the ability to link mutational data and structural features, and propose an explanation of what makes a good and bad RNA binding site in this case. In particular, we have underlined the importance of the formation of the T2 triplet (U40–A43–U66 ) and the M2 configuration for peptide binding, in agreement with experiment. While our model is reduced in size to allow efficient computation, we were able to discuss in detail the importance of U40, U41 and U63 in the RNA hairpin. When we were close to completing our study, the NMR structure published by Pham *et al.* (32), which we did not include in our simulations, confirmed our findings that the M2 configuration is the key to binding. Their results enable us to identify further similarities between our simulations and experimentally observed binding.

As a more general consideration, our study suggests that mutations, even of a single residue, have the potential for remodelling the structure in more subtle ways than just breaking base-pairs or shortening a link, but they can trigger cascade effects that cause the rearrangement of a large portion of the structure.

## Supplementary Material

gkz1071_Supplemental_File
