# Supplementary material for: Structural transitions in the RNA 7SK 5′ hairpin and their effect on HEXIM binding
Source: Nucleic Acids Res. 2019 Nov 16;48(1):373–89. doi: 10.1093/nar/gkz1071 (PMC7145557; doi:10.1093/nar/gkz1071)
Supplement: gkz1071_Supplemental_File [file gkz1071_supplemental_file.pdf]

Supplementary Material: Structural transitions in the RNA 7SK 5′ hairpin and their effect on HEXIM binding

K. Röder[1], G. Stirnemann[2], A. C. Dock-Bregeon[3], D. J. Wales *[1], and S. Pasquali †[4]

[1]*Department of Chemistry, University of Cambridge, Lensfield Road, Cambridge, CB2 1EW, UK,*

[2]*Laboratoire de Biochimie Théorique, IBPC, UPR CNRS 9080, 13 rue Pierre et Marie Curie, 75005 Paris, France,*

[3]*Laboratoire de Biologie Intégrative des Modèles Marins, UMR CNRS 8227, Sorbonne University, Station Biologique de Roscoff, 29680 Roscoff, France*

[4]*Laboratoire de Cristallographie et RMN Biologiques, UMR CNRS 8015, Paris Descartes University, 4 Avenue de l'observatoire, 75270 Paris, France*

S1 Molecular Dynamics simulations of HP1 24-87

In our first simulation study of HP1 [1] we considered the system with all bases neutrally charged. The hairpin was observed to be rather flexible. However, a careful analysis of the crystal structure, also confirmed by the recent NMR structure (Exp4), highlighted the presence of three protonated bases (C71, C75 and A77). We report here results of molecular dynamics (MD) simulations for the experimental structure Exp2 in a system with all neutral bases [1] and for a system where the bases C71, C74 and A77 are positively charged, as suggested by the crystal structures. MD simulations were performed for the fully solvated system and run for 200 ns. We observe that the protonated molecule adopts a straighter configuration with respect to the neutral model, and it fluctuates less (see Figure S1). This increased rigidity of the protonated model is also reflected by the RMSD and by the overall bend. The RMSD for the protonated molecule remains closer to the initial (experimental) structure, with values ranging from 2 to 4 Å . The RMSD for the neutral model has significantly higher values and larger fluctuations, ranging from 6 to 10 Å with respect to the crystal structure. We also analyse the overall bend of the molecule, as given by the angle formed by atoms P28-P48-P57. We find that the protonated model remains straighter (values closer to 180 degrees) and exhibits fewer fluctuations than the unprotonated model, for which the bend increases as the simulation progresses, with values ranging from the initial 160 degrees to as low as 100 degrees.

*dw34@cam.ac.uk

†samuela.pasquali@parisdescartes.fr

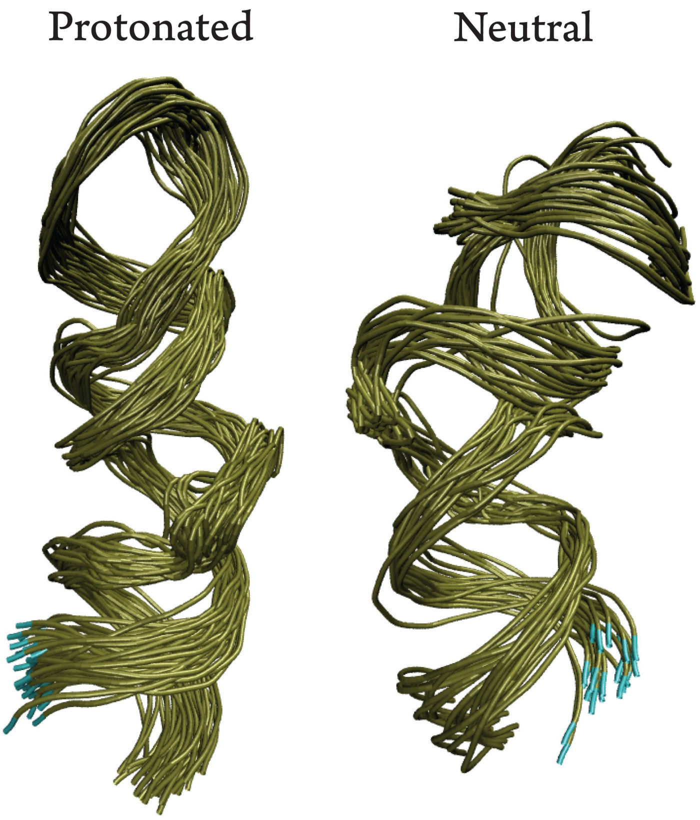

Figure S1: Comparison of the fluctuations of the experimental structure Exp2 for a fully atomistic MD simulation. Left: Superposition of simulated structures for the model with protonated C71, C74 and A77; Right: superposition of simulated structures for the model with all neutral bases.

S2 Molecular Dynamics Setup

S2.1 Equilibration

The following protocol was used for both standard MD simulations and for H-REX simulations. All the runs were performed using Gromacs 5.1.2.[2] Each RNA system was first neutralized with potassium cations, and then solvated in a 6 nm side cubic box containing a 0.2 M KCl solution. All bonds involving hydrogen atoms were maintained rigid using the LINCS algorithm.[3] Temperature was maintained at 300 K using the stochastic velocity rescaling algorithm with a time constant of 0.1 ps,[4] and pressure was fixed to 1 bar using the Parinello-Rahman barostat[5] with a time constant of 0.5 ps. A cut-off of 1.1 nm was employed for van der Waals and real-space electrostatics interactions, while long-range electrostatics was treated using PME[6] with a Fourier grid spacing of 1 Å^{-1}. All simulations were then equilibrated as follows: first, the potential energy was minimized until convergence; the solvent molecules were then relaxed for 80 ps at the target temperature, while the macromolecule was maintained fixed. The whole system was then heated to 300 K for 400 ps, and later equilibrated for another 400 ps at 300 K before subsequent production runs were performed.

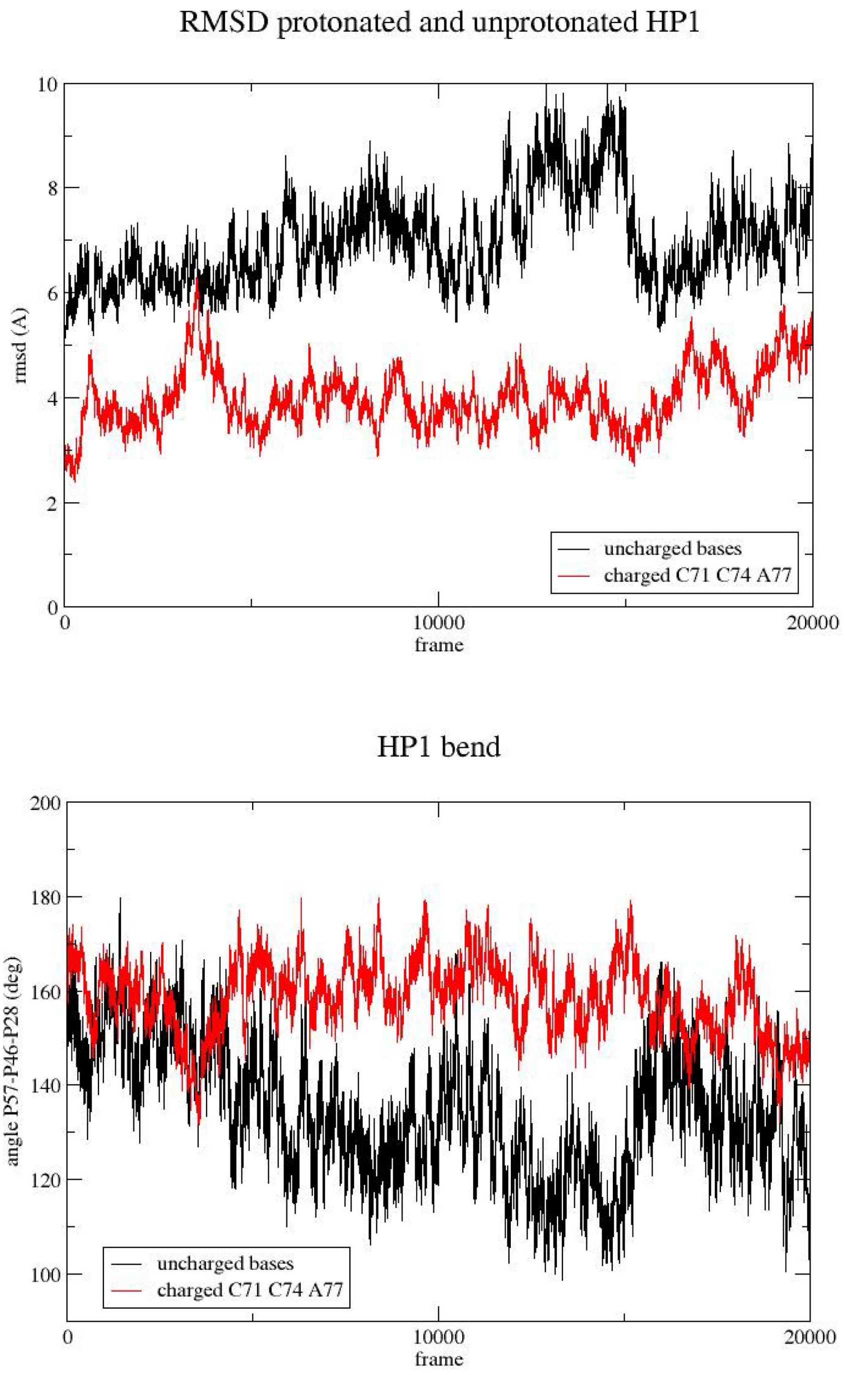

Figure S2: Comparison of the RMSD (top) and the overall bending (bottom) of the experimental structure Exp2 for a fully atomistic MD simulation of the protonated and the neutral system.

S2.2 Hamiltonian replica exchange simulations

For selected systems, enhanced sampling of the RNA conformational space was achieved using a Hamiltonian replica exchange scheme. The REST2[7–9] algorithm was employed, as it can be successfully applied to biomolecules in explicit solvent. Instead of performing simulations of several replicas with different physical temperatures, as in the more standard temperature

replica exchange approach, in the REST2 approach only the Hamiltonian of the biomolecule is rescaled, and the number of replicas thus scales with the biomolecule size, and not that of the entire simulation box. Details about this algorithm and its application to biomolecules can be found elsewhere.[7–9] Briefly, in a given replica n, all RNA-RNA interactions are rescaled by λ_n and all RNA-solvent interactions by $\sqrt{\lambda_n}$, with $0 < \lambda_n \leq 1$. In our REST2 setup, we employed 24 replicas and a distribution of λ_n such that for the n-th replica (from 1 to 24) $\lambda_n = (1/2)^{n/24}$. REST2 simulations were propagated for 200 ns using Gromacs 5.1.2 patched with Plumed 2.3.[10] Simulation details were similar to that described above for equilibrium MD trajectories, and exchanges between neighbouring replicas were attempted every 5 ps.

S3 Path sampling setup

S3.1 Energy landscape exploration

The exploration of the energy landscape within the computational potential energy landscape framework[11,12] aims to extract kinetic, thermodynamic and structural data via the construction of kinetic transition networks (KTNs)[13,14] using discrete path sampling (DPS).[15,16] The KTN is formed by discrete paths, a series of local minima, where each pair is connected by a transition state, here defined as a Hessian index 1 saddle point. Transition state candidates are located using the doubly-nudged[17] elastic band algorithm[18,19] (DNEB), and subsequent refinement of these candidate structures is achieved with hybrid eigenvector-following (HEF).[20,21] The local minima connected by a transition state are identified by following approximate steepest-descent pathways defined by the unique eigendirection of the transition state. For all optimisation procedures a customised L-BFGS minimisation is used with a convergence criterion on the RMS force of 10^{-6} kcal mol^{-1}.

Connections between the experimental structures were located using a modified Dijkstra-selector[22] to yield an initial database. This database was refined by removing artificially high energy barriers[23] and kinetic traps,[23] and the connectivity was improved using local sampling.[24] GPU acceleration was employed throughout to speed up the calculations.[25–27] Free energies were calculated using the harmonic superposition approach[28] in combination with recursive regrouping.[29]

S3.2 Starting structures and force fields

For the native sequence, three different configurations are available from the protein data bank: identifier 5LYU for the two crystal structures[1] and 5IEM for the NMR structure.[30] The mutants were created from these structures using the LEAP package in AMBER12.[31] The ff99[32] force field with the Barcelona α/γ backbone modification[33] and the χ modification tuned for RNA[34,35] was employed with implicit solvent ($igb = 2$). To study HEXIM binding, the properly symmetrised ff14SB[36] force field was used for the ARM peptide, and the sequence given in the main text was capped.

S3.3 Probing binding to ARM

To test the binding of ARM to the 7SK motif, low energy structures from the energy landscape of the wild type were used, and ARM was added to these configurations using PyMOL.[37] We chose a low energy M1 configuration and two low energy M2 configurations, one with U41 pointing outwards and the other with it pointing inwards, as well as one extended structure. The potential energy was optimised using basin-hopping global optimisation with fixed coordinates for the RNA within the local rigid body framework,[38,39] followed by DPS. The lowest energy structures were then used to run MD simulations in explicit solvent without restraints. An

octahedral solvent box with TIP3P water and a minimum distance of 14 Å between the RNA – peptide complex and the box edge was employed. The system was heated to 300 K with harmonic restraints on the RNA and peptide, and the restraints were slowly reduced. The pressure was equilibrated in the NPT ensemble with initial constraints on the RNA – peptide complex, which were subsequently removed. After equilibration the production runs were performed in the NVT ensemble at 300 K with a time step of 2 fs. A number of short 50 ns trajectories were run, as well as longer trajectories of 500 ns.

S4 Wild type complementary figures

We report here more detailed figures from the analysis of both path sampling and MD simulations for the wild type sequences.

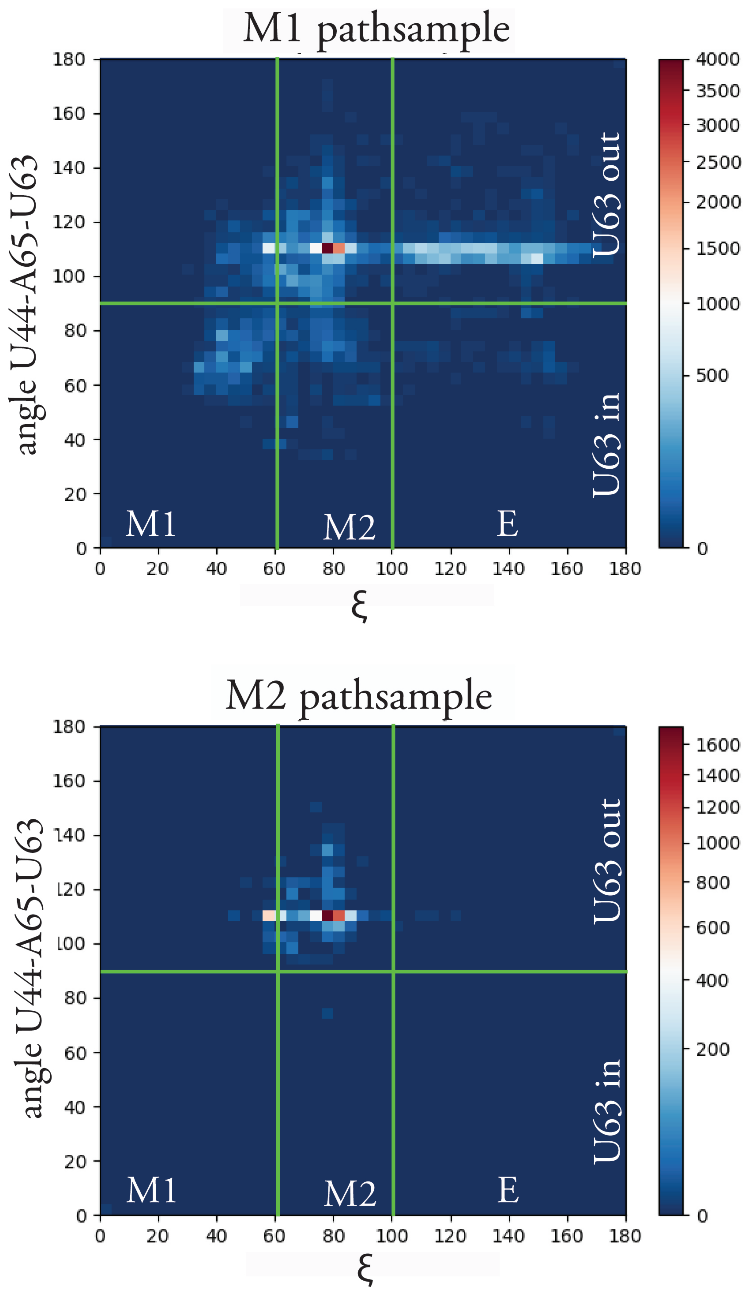

Figure S3: Projection of the path sampling database of native sequences onto the variables ξ and the angle U44-A65-U63, describing the inward or outward position of U63. Structures included in heat-plots are the minima accessible from M1 or M2 with a threshold of 15 kcal/mol from the bottom of the respective free energy funnels. This threshold means we only include minima that can be reached from the respective structure on a time of milliseconds.[40] The projection only represents the density of minima, and no occupation probabilities or energies are used to weight the result.

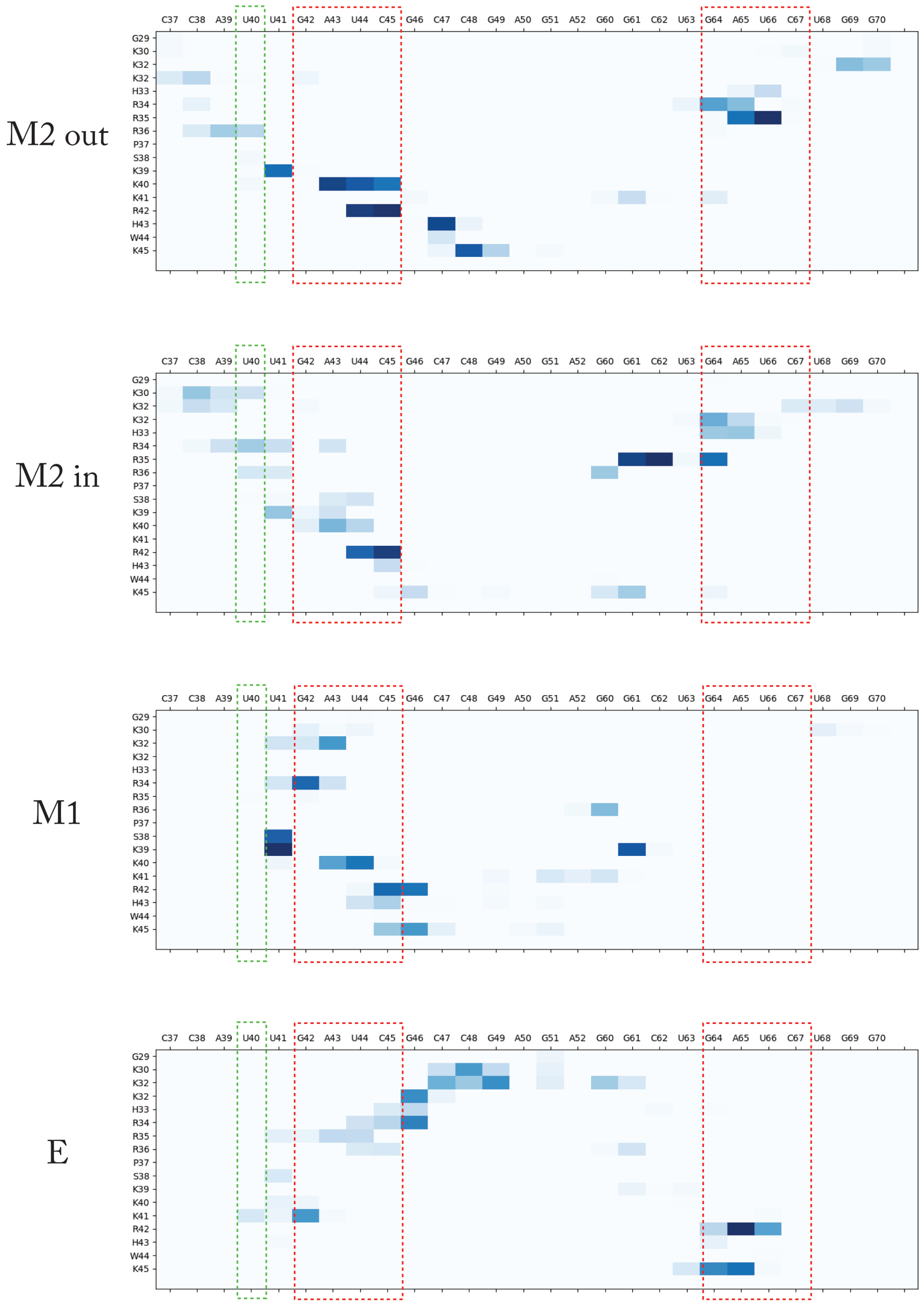

Figure S4: Hydrogen bonds formed between peptide residues and RNA. We measure the presence of a bond as a percentage over the full MD trajectory (dark blue 100%, white 0%). Dashed boxes highlight the interactions of U40 (green) and of the GAUC/GAUC motif (red).

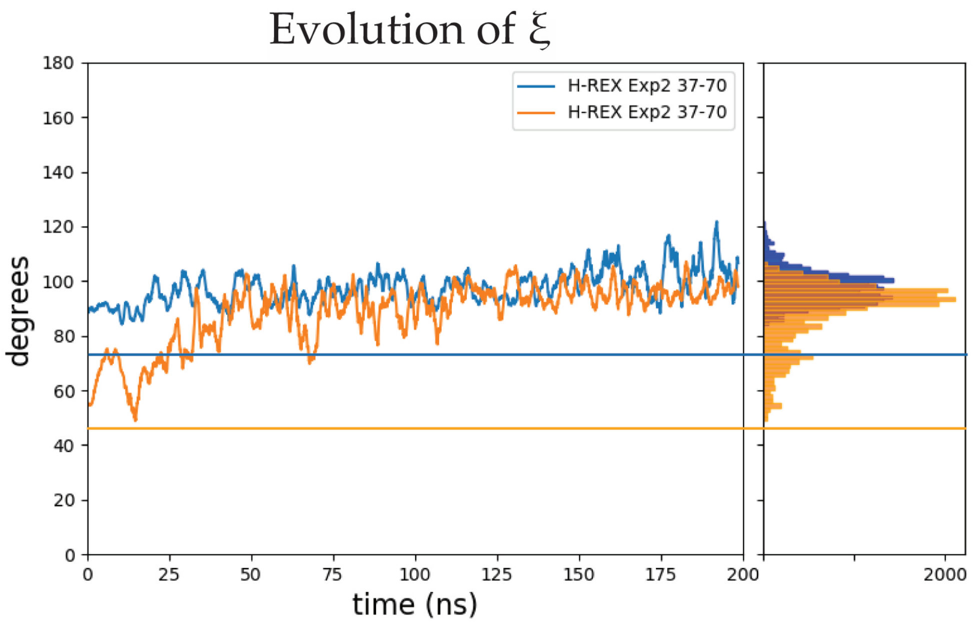

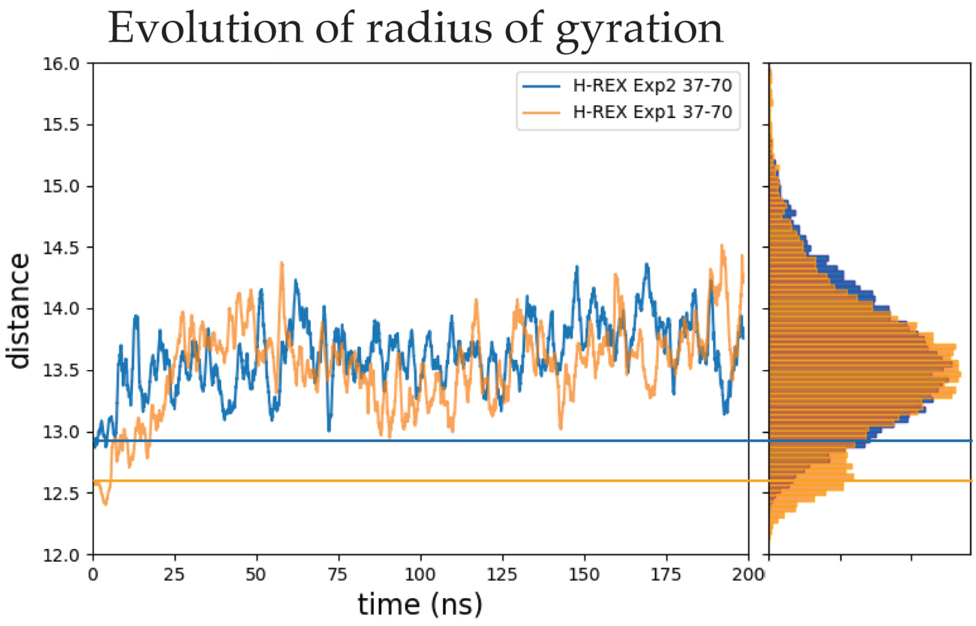

Figure S5: Results from H-REX for the wild type showing the behavior of the variable ξ, and the radius of gyration, smoothed with a running window of 150 frames. The straight horizontal lines indicate the values of ξ and of the radius of gyration measured in the experimental structures, in blue for the simulations initiated by Exp2 and in orange by Exp1.

S5 Mutations: Data and figures

Here, we present more detailed results for the mutants. Figures S6, S7 and S8 show the corresponding energy landscapes. In Table S1 a summary of structural descriptors is given, with labels indicating their position in the disconnectivity graphs provided.

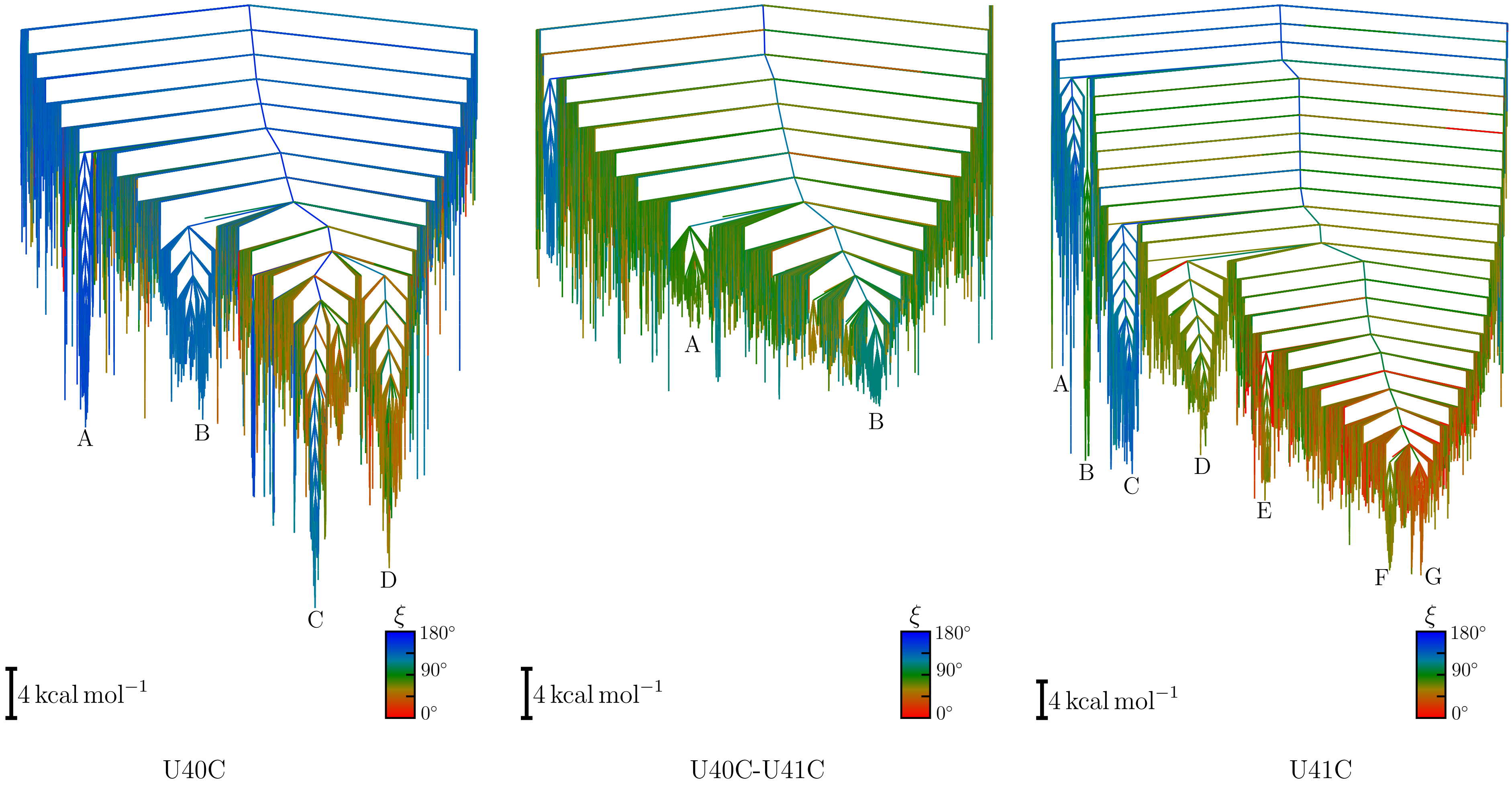

Figure S6: Potential energy disconnectivity graphs for the U40C, U40C+U41C and U41C mutants of the RNA 7SK HP1 hairpin. The labels A to G correspond to distinct structural ensembles, which are characterised in detail in Table S1. This figure otherwise corresponds to Figure 7 in the main text.

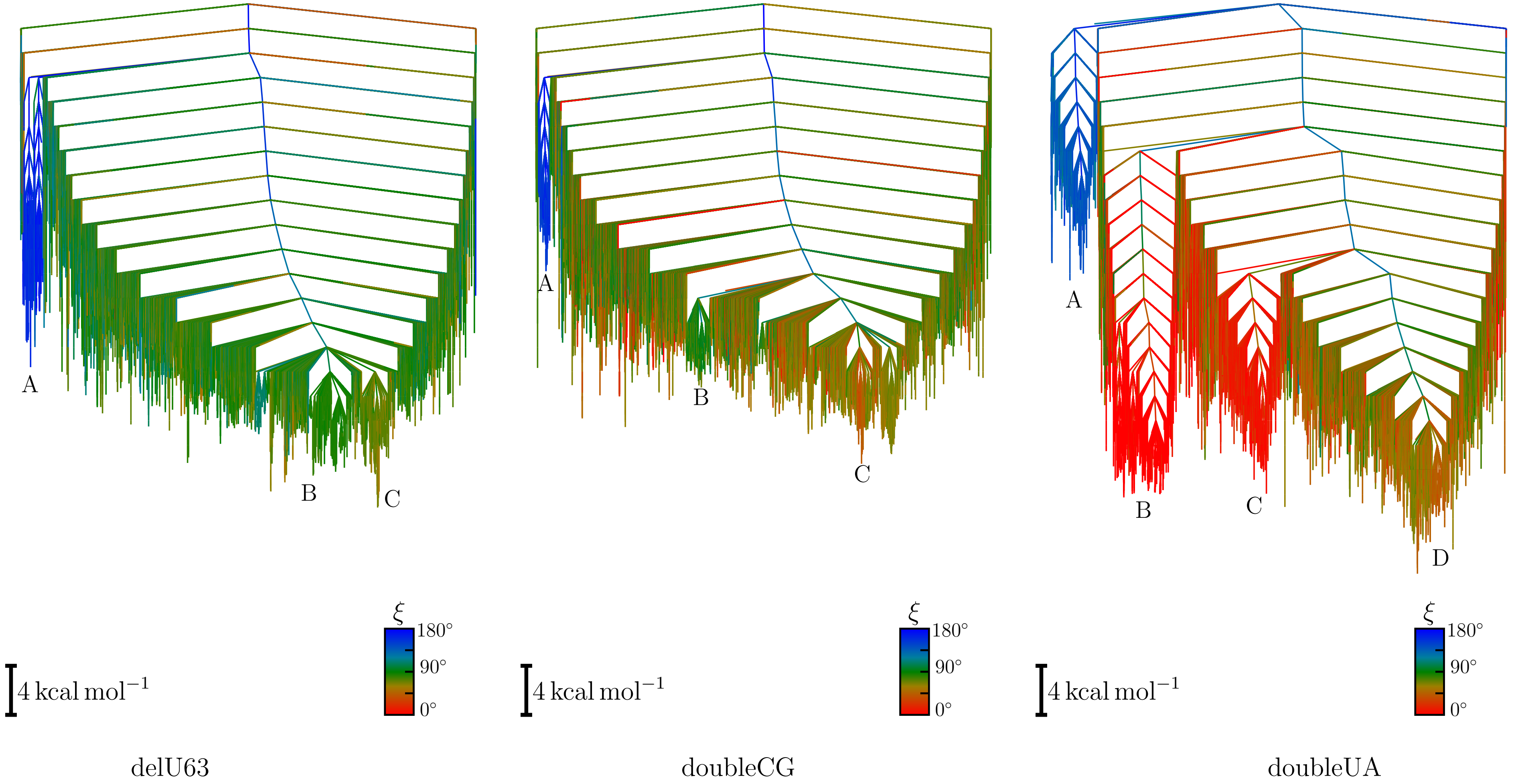

Figure S7: Potential energy disconnectivity graphs for the delU63, the doubleCG and doubleUA mutants of the RNA 7SK HP1 hairpin. While the deletion of U63 still allows for the formation of some M2 structures, the changes in the 7SK motif in the doubleUA and doubleCG mutants, which destabilise the T2 triplet, lead to an increase in the number of extended structures. The labels A to D correspond to distinct structural ensembles, which are characterised in detail in Table S1.

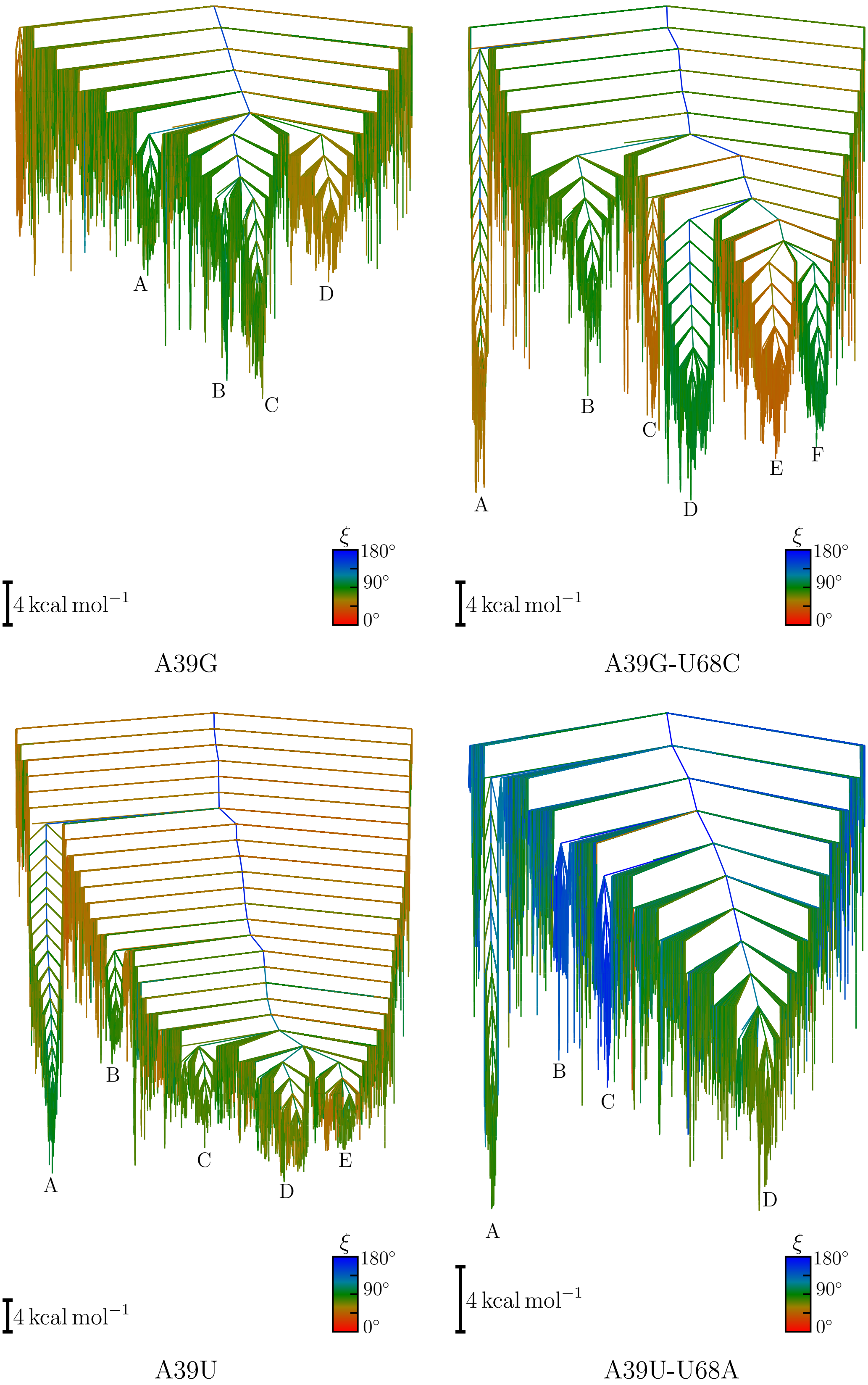

Figure S8: Disconnectivity graphs for the potential energy landscapes of the A39G, A39G-U68C, A39U and A39U-U68A mutants. The labels A to F correspond to distinct structural ensembles, which are characterised in detail in Table S1.

mutant	Label	ΔV_{gmin} (kcal/mol)	$\Delta E_{\mathrm{funnel}}$ (kcal/mol)	classification M1/M2/E (%)	U63 in/out (%)	N_{hb} U40 (average)	N_{hb} U41 (average)	U40-X68 (%)
A39G		16	22	100/0/0 (M1)	100/0	2.8	1.6	0
	A	12	16	0/50/50 (M2/E)	82/18	1.5	1.2	0
		10	10	0/100/0 (M2)	87/13	1.9	0.0	0
	B	2	20	0/93/7 (M2)	0/100	1.1	1.4	0
	C	0	22	0/100/0 (M2)	99/1	1.7	0.0	0
	D	12	16	0/0/100 (E)	100/0	1.9	2.0	0
A39G-U68C	A	0	42	0/95/5 (M2)	87/13	3.4	0.0	0
	B	8	26	0/100/0 (M2)	85/15	1.6	0.0	0
	C	6	22	100/0/0 (M1)	100/0	2.7	1.7	0
	D	0	28	0/99/1 (M2)	10/90	1.0	1.9	0
	E	4	20	96/0/4 (M1)	100/0	1.1	1.9	0
	F	4	20	0/93/7 (M2)	90/10	0.9	1.9	0
A39U		14	46	99/0/1 (M1)	100/0	1.9	1.3	0
	A	2	46	3/39/58 (E)	37/63	1.5	1.7	0
	B	16	16	0/100/0 (M2)	100/0	1.0	0.0	0
	C	4	14	0/0/100 (Ek)	73/27	1.1	1.6	61
	D	0	18	0/5/95 (Ek)	100/0	1.9	1.0	64
	E	4	14	0/0/100 (Ek)	99/1	1.6	1.1	41
A39U-U68A	A	0	29	0/84/16 (M2)	2/98	1.1	1.3	0
	B	9	16	47/0/53 (M1/E)	100/0	1.8	1.6	0
	C	8	15	92/0/8 (M1)	100/0	2.6	1.5	0
	D	0	17	0/94/6 (M2)	86/14	1.3	1.0	0
delU63	A	12	26	99/0/1 (M1)	-	2.7	2.0	0
	B	3	9	89/0/10 (M1)	-	1.9	2.6	0
	C	0	12	0/3/97 (Ek)	-	2.7	2.1	98
doubleCG	A	16	18	100/0/0 (M1)	100/0	4.4	2.2	0
	B	6	10	0/88/12 (M2)	0/100	1.9	1.9	0
	C	0	12	0/0/100 (Ek)	0/100	2.4	2.1	99
doubleUA	A	24	23	99/0/1 (M1)	58/41	3.7	2.2	0
	B	7	16	0/6/94 (Ek)	0/100	2.3	1.6	0
	C	7	20	0/0/100 (E)	0/100	2.7	1.3	90
	D	0	27	5/23/72 (E)	0/100	2.1	2.0	65
U40C	A	14	24	96/0/4 (M1)	100/0	2.4	1.4	0
	B	14	18	0/63/37 (M2)	84/16	2.6	0.4	0
	C	0	24	0/0/100 (E)	94/6	1.5	2.2	44
		12	12	0/0/100 (E)	74/26	0.9	1.6	97
	D	2	26	2/12/86 (E)	2/98	0.9	1.2	72
U40C-U41C		12	17	94/0/6 (M1)	100/0	3.6	2.8	0
	A	6	11	0/100/0 (M2)	100/0	1.6	1.0	0
	B	0	17	0/20/80 (E)	7/93	3.3	2.6	31
U41C	A	13	44	59/0/41 (M1/E)	97/3	2.0	1.9	0
	B	13	44	0/6/94 (Ek)	0/100	2.2	1.7	0
	C	11	30	84/0/10 (M1)	100/0	2.6	1.7	0
	D	13	24	0/100/0 (M2)	49/51	1.0	0.0	0
	E	8	19	0/0/100 (E)	81/19	0.9	1.8	86
	F	1	14	0/98/2 (M2)	0/100	0.9	1.4	0
	G	0	15	0/0/100 (E)	0/100	0.9	1.8	85

Table S1: Summary of discrete path sampling results for all mutants. For distinct funnels on the energy landscapes shown in Fig. S6-S8 we report the potential energy difference between the global minimum and funnel bottom, ΔV_{gmin}, the depth of the funnel (potential energy from the lowest minimum in the funnel to the lowest energy transition state connecting it to the global minimum), $\Delta E_{\mathrm{funnel}}$, the classification as M1, M2 or E structures, indicating whether we detect the artificial kink of the lowest stem folding back on the rest of the structure (Ek), the position of U63, the average number of hydrogen bonds made by the nucleotides 40 and 41, including bonds made by the base as well as by the phosphate group, and the percentage of structures exhibiting a U40-X68 base pairing.

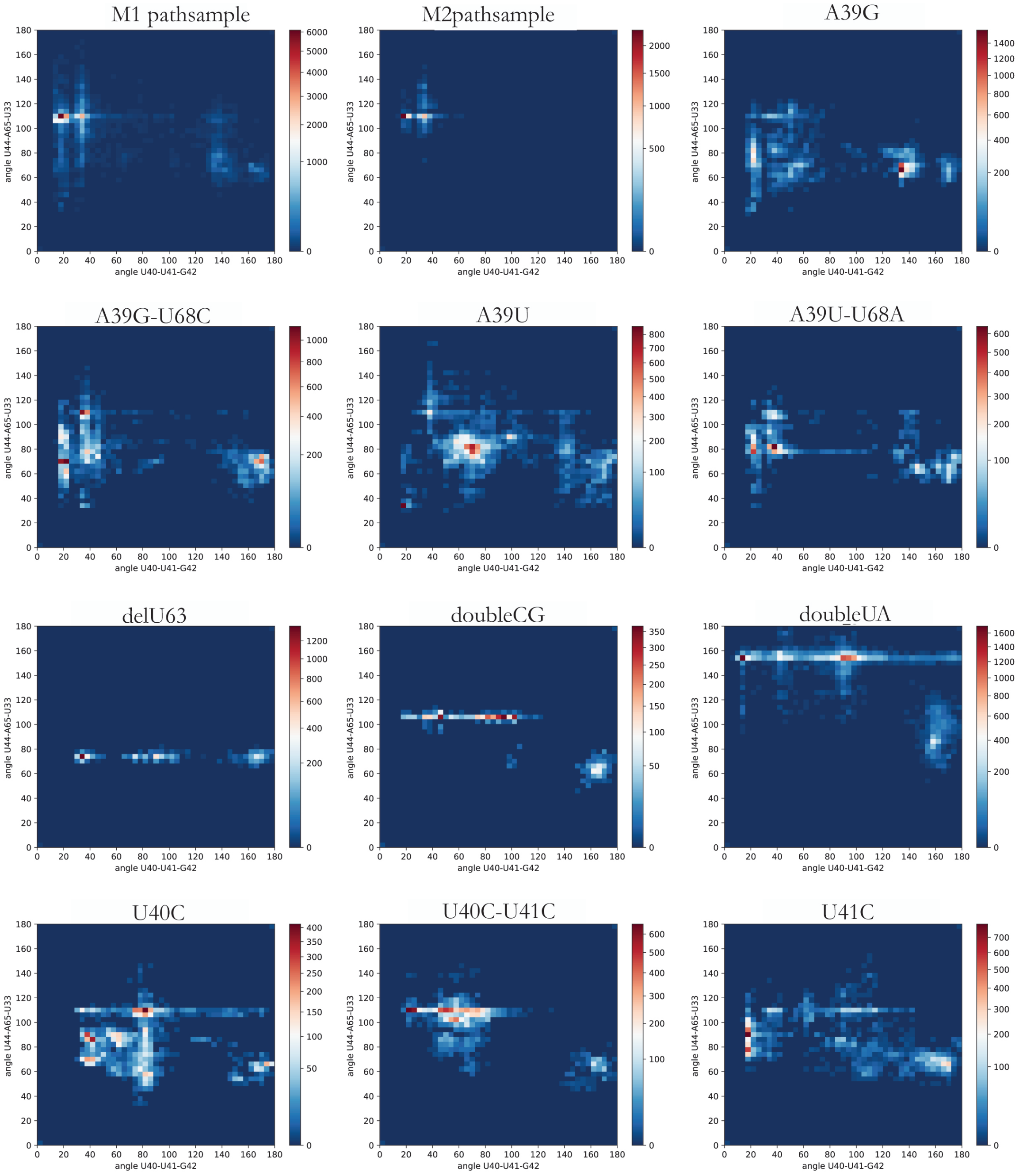

Figure S9: Projection of the path sampling database of native and mutated sequences onto the variables describing the behaviour of U40 and U41 with respect to the triplets T1 and T2 (T2 angle U40-U41-G42 $\leq$ 40, T1: angle U40-U41-G42 $\geq$ 160) and the behavior our U63 (inward: angle U44-A65-U63 $\leq$ 90, angle outward: U44-A65-U63 $\geq$ 90). These projections simply present densities of minima with respect to the two chosen parameters. These plots are not weighted by occupation probabilities or energies.

M1 (ns)	M2 (ns)
19.5	4.9
3.8	8.0
4.4	9.1
25.4	12.1
17.1	3.6
5.6	4.6
12.6	15.9
16.7	3.2
11.9	11.8
12.2	17.4
21.7	9.8
29.4	9.3
8.0	5.0
31.5	13.1
10.0	6.0
9.3	4.5
6.7	5.4
30.2	7.5
24.8	13.4
7.5	6.4
6.2	3.9
5.9	7.7
11.3	9.2
9.7	8.8
14.23 ±8.74	*8.36 ±3.94*

Table S2: Temporal trajectories: autocorrelation times of the variable ξ from the 24 reconstructed continuous trajectories of the H-REX for the wild system, starting from either M1 or M2 as initial configurations. The last line gives the average and the standard deviation over all replicas.

S6 Statistical analysis of H-REX

The output of H-REX simulations are trajectories for a fixed value of λ (λ-trajectories), therefore subject to the exchanges of replicas. Using PLUMED, we reconstructed the 24 temporal trajectories, which are continuous trajectories for a given molecule, subject to a Hamiltonian of varying intensity. In our analysis we focused on the behavior of the variable ξ, discriminating between the three families of configurations, M1, M2 and E.

From the temporal trajectories we computed the autocorrelation time for ξ for the simulations starting from M1 and from M2 (Table S2). The overall simulation time of 200 ns is large compared to the characteristic times of the simulations, which average between 8 and 14 ns. We observe in some examples that in a given temporal trajectory ξ exhibits several transitions between values characteristic of M1, M2 and E (Fig. S10)

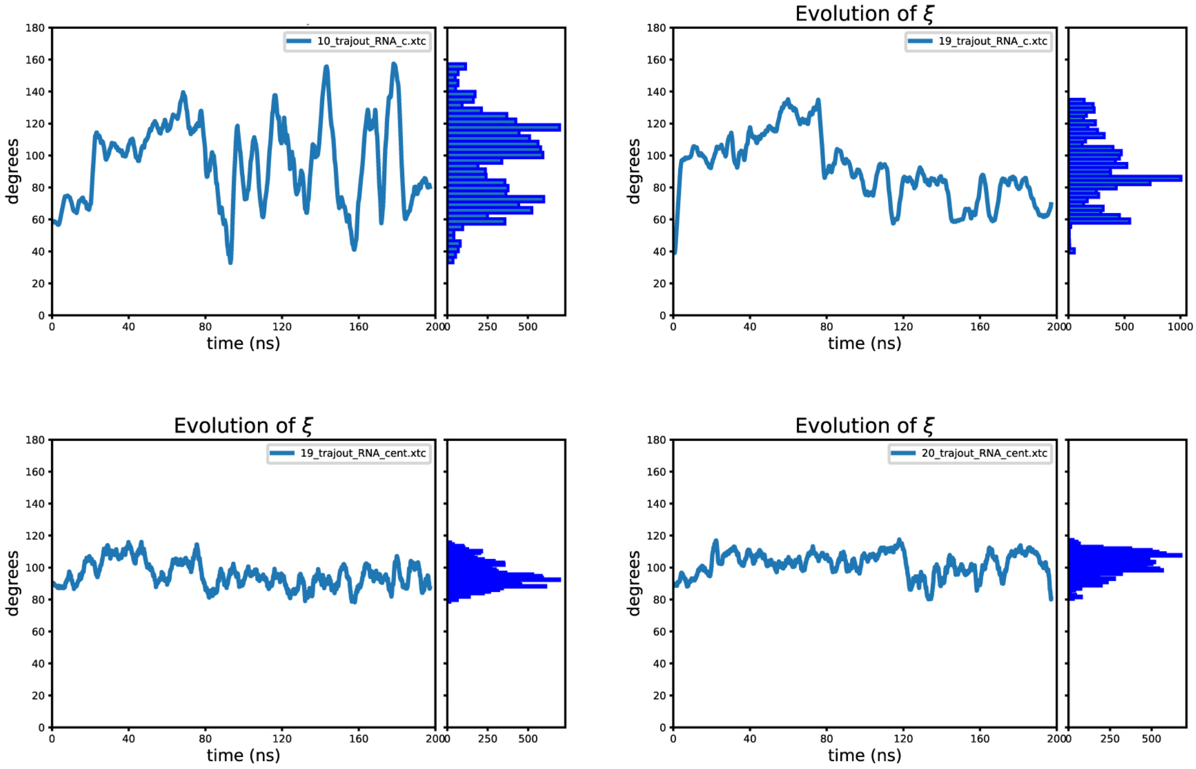

Figure S10: Temporal trajectories: evolution of ξ for two trajectories starting from M1 (top) and two trajectories starting from M2 (bottom).

A block analysis for the λ=1 trajectory (full Hamiltonian) exhibits a shift in population from M1 to M2 for simulations starting from M1, and a more stable M2 population for simulations starting from M2 (Fig. S11). From the λ-trajectories we computed the populations of M1, M2 and E for each value of λ (Table S3) focusing on the second half of the trajectories (100 ns to 200 ns) to avoid the initial transient behavior of the simulations. As expected, as the value of λ decreases, local structures are lost, ξ assumes a broad distribution, and results are similar for the simulations starting from Exp1 and from Exp2.

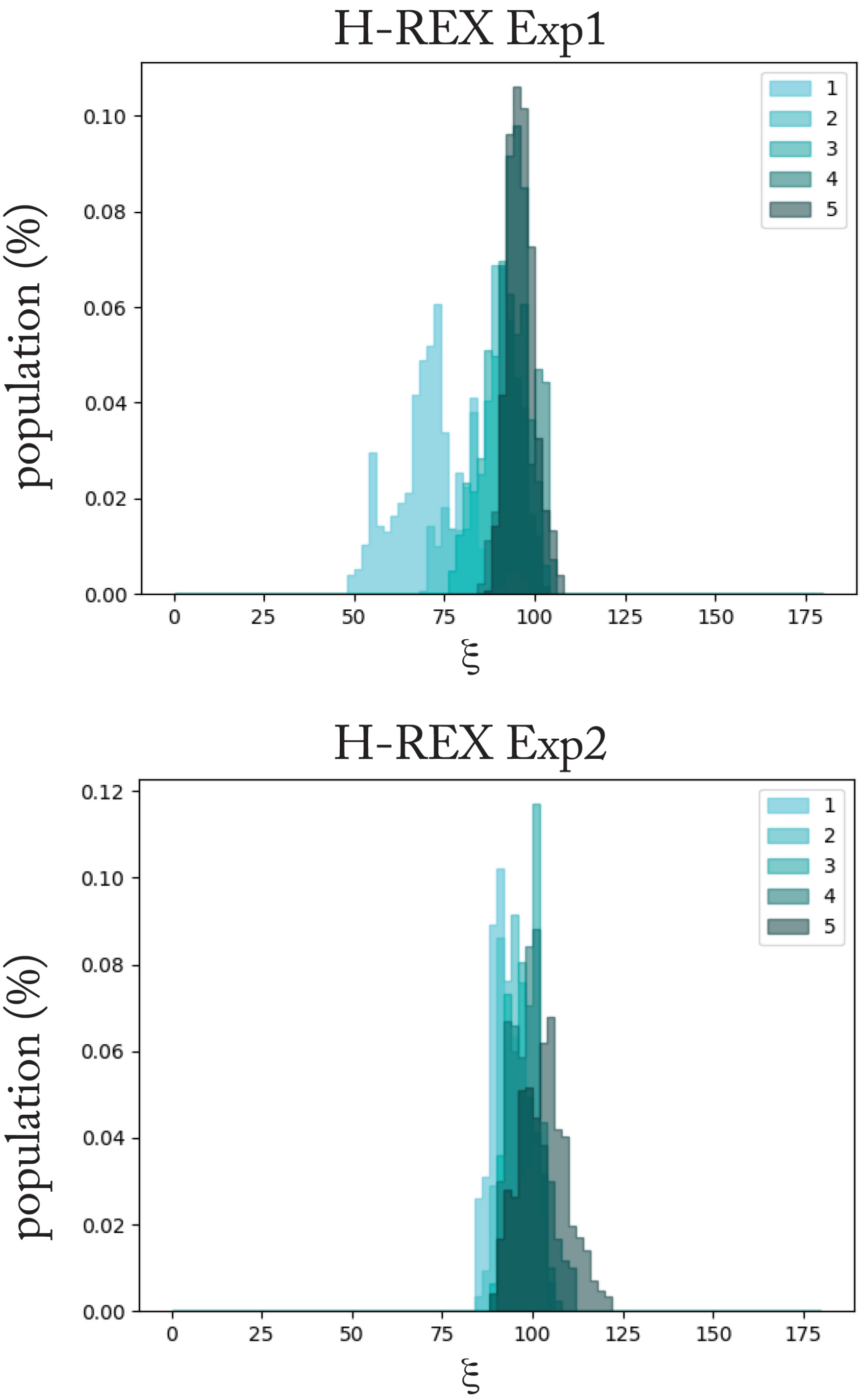

Figure S11: Block analysis of the distribution of ξ for the full Hamiltonian trajectories starting from M1 (top) and M2 (bottom). The trajectory has been divided into 5 blocks of 40 ns each.

Replica		Exp1 simulations			Exp2 simulations		
n	λ	M1	M2	E	M1	M2	E
0	1.00	0.06	0.68	0.27	0.00	0.64	0.36
1	0.97	0.06	0.66	0.27	0.00	0.63	0.37
2	0.94	0.07	0.66	0.27	0.00	0.63	0.37
3	0.92	0.08	0.66	0.26	0.00	0.66	0.34
4	0.89	0.08	0.65	0.27	0.00	0.66	0.34
5	0.87	0.08	0.63	0.29	0.00	0.66	0.34
6	0.84	0.09	0.61	0.30	0.00	0.65	0.35
7	0.82	0.09	0.58	0.32	0.00	0.62	0.38
8	0.79	0.09	0.57	0.34	0.00	0.60	0.40
9	0.77	0.10	0.54	0.37	0.00	0.56	0.43
10	0.75	0.11	0.52	0.37	0.00	0.51	0.48
11	0.73	0.13	0.49	0.38	0.01	0.47	0.52
12	0.71	0.11	0.48	0.41	0.01	0.43	0.57
13	0.69	0.12	0.45	0.43	0.01	0.39	0.61
14	0.67	0.10	0.44	0.45	0.01	0.37	0.63
15	0.65	0.09	0.44	0.48	0.01	0.34	0.64
16	0.63	0.09	0.42	0.48	0.02	0.33	0.65
17	0.61	0.12	0.42	0.47	0.05	0.32	0.64
18	0.59	0.14	0.41	0.45	0.13	0.34	0.52
19	0.58	0.15	0.40	0.45	0.15	0.36	0.49
20	0.56	0.16	0.37	0.46	0.16	0.36	0.48
21	0.55	0.18	0.38	0.45	0.17	0.37	0.47
22	0.53	0.20	0.36	0.43	0.19	0.36	0.45
23	0.51	0.22	0.36	0.42	0.22	0.34	0.44

Table S3: λ-trajectories: populations of the families M1, M2 and E computed from the cutoffs defined previously (M1: $\xi < 60^o$, M2: $60^o \leq \xi < 100^o$, E: $\xi \geq 100^o$) in the second half of the trajectory.

References

[S1] Martinez-Zapien, D.; Legrand, P.; McEwen, A. G.; Proux, F.; Cragnolini, T.; Pasquali, S.; Dock-Bregeon, A.-C. *Nucleic Acids Res.* **2017**, *45*, 3568–3579.

[S2] Berendsen, H.; van der Spoel, D.; van Drunen, R. *Comput. Phys. Commun.* **1995**, *91*, 43 – 56.

[S3] Hess, B.; Bekker, H.; Berendsen, H.; Fraaije, J. *J. Comp. Chem.* **1997**, *18*, 1463–1472.

[S4] Bussi, G.; Donadio, D.; Parrinello, M. *J. Chem. Phys.* **2007**, *126*, 014101.

[S5] Parrinello, M.; Rahman, A. *Phys. Rev. Lett.* **1980**, *45*, 1196–1199.

[S6] Darden, T.; York, D.; Pedersen, L. *J. Chem. Phys.* **1993**, *98*, 10089–10092.

[S7] Wang, L.; Friesner, R. A.; Berne, B. J. *J. Phys. Chem. B* **2011**, *115*, 9431–9438.

[S8] Bussi, G. *Mol. Phys.* **2014**, *112*, 379–384.

[S9] Stirnemann, G.; Sterpone, F. *J. Chem. Theory Comput.* **2015**, *11*, 5573–5577.

[S10] Tribello, G. A.; Bonomi, M.; Branduardi, D.; Camilloni, C.; Bussi, G. *Comp. Phys. Commun.* **2014**, *185*, 604 – 613.

[S11] Wales, D. J. *Energy Landscapes*; Cambridge University Press: Cambridge, 2003.

[S12] Joseph, J. A.; Röder, K.; Chakraborty, D.; Mantell, R. G.; Wales, D. J. *Chem. Commun.* **2017**, *53*, 6974–6988.

[S13] Noé, F.; Fischer, S. *Curr. Opin. Struc. Biol.* **2008**, *18*, 154–162.

[S14] Wales, D. J. *Curr. Opin. Struc. Biol.* **2010**, *20*, 3–10.

[S15] Wales, D. J. *Mol. Phys.* **2002**, *100*, 3285–3305.

[S16] Wales, D. J. *Mol. Phys.* **2004**, *102*, 891–908.

[S17] Trygubenko, S. A.; Wales, D. J. *J. Chem. Phys.* **2004**, *120*, 2082–2094.

[S18] Henkelman, G.; Jónsson, H. *J. Chem. Phys.* **2000**, *113*, 9978–9985.

[S19] Henkelman, G.; Uberuaga, B. P.; Jónsson, H. *J. Chem. Phys.* **2000**, *113*, 9901–9904.

[S20] Munro, L. J.; Wales, D. J. *Phys. Rev. B* **1999**, *59*, 3969–3980.

[S21] Henkelman, G.; Jónsson, H. *J. Chem. Phys.* **1999**, *111*, 7010–7022.

[S22] Carr, J. M.; Trygubenko, S. A.; Wales, D. J. *J. Chem. Phys.* **2005**, *122*, 234903.

[S23] Strodel, B.; Whittleston, C. S.; Wales, D. J. *J. Am. Chem. Soc.* **2007**, *129*, 16005–16014.

[S24] Röder, K.; Wales, D. J. *J. Am. Chem. Soc.* **2018**, *140*, 4018–4027.

[S25] Götz, A. W.; Williamson, M. J.; Xu, D.; Poole, D.; Le Grand, S.; Walker, R. C. *J. Chem. Theory Comput.* **2012**, *8*, 1542–1555.

[S26] Salomon-Ferrer, R.; Götz, A. W.; Poole, D.; Le Grand, S.; Walker, R. C. *J. Chem. Theory Comput.* **2013**, *9*, 3878–3888.

[S27] Mantell, R. G.; Pitt, C. E.; Wales, D. J. *J. Chem. Theory Comput.* **2016**, *12*, 6182–6191.

[S28] Strodel, B.; Wales, D. J. *Chem. Phys. Lett.* **2008**, *466*, 105–115.

[S29] Carr, J. M.; Wales, D. J. *J. Phys. Chem. B* **2008**, *112*, 8760–8769.

[S30] Bourbigot, S.; Dock-Bregeon, A.-C.; Eberling, P.; Coutant, J.; Kieffer, B.; Lebars, I. *RNA* **2016**, *22*, 1844–1858.

[S31] AMBER12. Case, D.A. et al.: University of California, San Francisco, 2012.

[S32] Wang, J.; Cieplak, P.; Kollman, P. A. *J. Comput. Chem.* **2000**, *21*, 1049–1074.

[S33] Pérez, A.; Marchán, I.; Svozil, D.; Sponer, J.; Cheatham, T. E.; Laughton, C. A.; Orozco, M. *Biophys. J.* **2007**, *92*, 3817–3829.

[S34] Banáš, P.; Hollas, D.; Zgarbová, M.; Jurečka, P.; Orozco, M.; Cheatham, T. E.; Šponer, J.; Otyepka, M. *J. Chem. Theory Comput.* **2010**, *6*, 3836–3849.

[S35] Zgarbová, M.; Otyepka, M.; Šponer, J.; Mládek, A.; Banáš, P.; Cheatham, T. E.; Jurečka, P. *J. Chem. Theory Comput.* **2011**, *7*, 2886–2902.

[S36] Maier, J. A.; Martinez, C.; Kasavajhala, K.; Wickstrom, L.; Hauser, K. E.; Simmerling, C. *J. Chem. Theory Comput.* **2015**, *11*, 3696–3713.

[S37] The PyMOL Molecular Graphics System, Version 1.6.x. Schrödinger, LLC: New York, 2013.

[S38] Rühle, V.; Kusumaatmaja, H.; Chakrabarti, D.; Wales, D. J. *J. Chem. Theory Comput.* **2013**, *9*, 4026–4034.

[S39] Kusumaatmaja, H.; Whittleston, C. S.; Wales, D. J. *J. Chem. Theory Comput.* **2012**, *8*, 5159–5165.

[S40] Bothe, J. R.; Nikolova, E. N.; Eichhorn, J.; Chugh, J.; Hansen, A. L.; Al-Hashimi, H. M. *Nat. Methods* **2011**, *8*.
